# High-level dietary cadmium exposure is associated with global DNA hypermethylation in the gastropod hepatopancreas

**DOI:** 10.1371/journal.pone.0184221

**Published:** 2017-09-06

**Authors:** Dragos V. Nica, Cristina Popescu, George A. Draghici, Florina-Maria Andrica, Ionela A. Privistirescu, Iosif I. Gergen, Reinhard Stöger

**Affiliations:** 1 Faculty of Pharmacy, "Victor Babes" University of Medicine and Pharmacy, Timisoara, RO, P-ta Murgu Eftimie 2, Romania; 2 Institute of Life Sciences, "Vasile Goldis" Western University of Arad, Arad, RO, Romania; 3 Faculty of Pharmacy, "Vasile Goldis" Western University of Arad, Arad, RO, Romania; 4 Faculty of Medicine, "Victor Babes" University of Medicine and Pharmacy, Timisoara, RO, P-ta Murgu Eftimie 2, Romania; 5 Faculty of Food Processing Technology, Banat’s University of Agricultural Sciences and Veterinary Medicine "King Michael I of Romania" from Timisoara, Timisoara, RO, Calea Aradului 119, Romania; 6 School of Biosciences, University of Nottingham, Sutton Bonington Campus, Leicestershire, United Kingdom; University of Louisville School of Medicine, UNITED STATES

## Abstract

5-methylcytosine (5mC) is a key epigenetic mark which influences gene expression and phenotype. In vertebrates, this epigenetic mark is sensitive to Cd exposure, but there is no information linking such an event with changes in global 5mC levels in terrestrial gastropods despite their importance as excellentecotoxicological bioindicators of metal contamination. Therefore, we first evaluated total 5mC content in DNA of the hepatopancreas of adult *Cantareus aspersus* with the aim to determine whether this epigenetic mark is responsive to Cd exposure. The experiment was conducted under laboratory conditions and involved a continuous exposure, multiple dose- and time-point (14, 28, and 56 days) study design. Hepatopancreas cadmium levels were measured using Flame Atomic Absorption Spectrometry and the percentage of 5-mC in samples using an ELISA-based colorimetric assay. Snail death rates were also assessed. Our results, for the first time, reveal the presence of 5mC in *C*. *aspersus* and provide evidence for Cd-induced changes in global 5mC levels in DNA of gastropods and mollusks. Although less sensitive than tissue accumulation, DNA methylation levels responded in a dose- and time-dependent manner to dietary cadmium, with exposure dose having a much stronger effect than exposure duration. An obvious trend of increasing 5mC levels was observed starting at 28 days of exposure to the second highest dose and this trend persisted at the two highest treatments for close to one month, when the experiment was terminated after 56 days. Moreover, a strong association was identified between Cd concentrations in the hepatopancreas and DNA methylation levels in this organ. These data indicate an overall trend towards DNA hypermethylation with elevated Cd exposure. No consistent lethal effect was observed, irrespective of time point and Cd-dosage. Overall, our findings suggest that the total 5mC content in DNA of the hepatopancreas of land snails is responsive to sublethal Cd exposure and give new insights into invertebrate environmental epigenetics.

## Introduction

Trace metal contamination in natural environments is a major societal challenge that intimately connects ecological sustainability with food security and human health [1]. Cadmium (Cd) is a high-profile example of a hazardous trace metal despite being found naturally in the Earth's crust at an abundance of 0.1–0.5 milligrams per kilogram dry weight (mg/kgd. wt). It is ranked as a priority pollutant worldwide due to the very long biological half-life (10–30 years), substantive accumulation along the terrestrial food chains, and toxicity at concentrations one tenth that of lead (Pb), mercury (Hg), aluminum (Al), or nickel (Ni) [2,3]. Beside the kidneys, the hepatic tissue functions as the primary endpoint of Cd accumulation in higher bilaterians, such as vertebrates or mollusks [3]. Once accumulated, cadmium affects our bodies at multiple levels, including the networks shaping tissue and cell-type specific gene expression and the underlying epigenome [4]. Methylation of the genome, which encompasses the addition of methyl (-CH_3_) groups to DNA, is a key epigenetic mechanism involved in regulating gene expression. In particular, methylation at the 5-carbon of the cytosine ring (5-methylcytosine, 5mC) is the best-characterized DNA modification and is primarily associated with stable, long-term transcriptional silencing [5]. In gastropods, the presence of 5mC as a key component of the epigenetic system has been already demonstrated for the sea hare *Aplysia californica* (Cooper,1863) [6], the freshwater snail *Biomphalaria glabrata* (Say, 1818) [7], the mud snail *Zeacumantus subcarinatus* (Sowerby II, 1855) [8], and the bladder snail *Physella acuta* (Draparnaud, 1805) [9,10]. There is also compelling evidence for the presence of 5mC in the genome of the land snail *Helix pomatia* (Linnaeus, 1758) [11]. However,there is no relevant epigenetic information available concerning other terrestrial gastropods that are commonly used as ecotoxicological study systems, such as the brown garden snail*Cantareus aspersus* (Müller, 1774)[12–16]or the grove snail *Cepaea nemoralis* (Linnaeus, 1758)[17,18].

An altered DNA methylation status resulting from exposure to chemicals including arsenic (As) and zinc (Zn) has been described in ecologically relevant invertebrate species, such as annelids (segmented worms) and crustaceans [19–21]. Currently available data regarding mollusks are, however, scarce andrather inconclusive. To our knowledge, only two studies have investigated the impact of chemicals on global, genome-wide DNA methylation levels in gastropods and both of them evaluated the epigenetic effects of vinclozolin (VZ)–an organic compound–using the freshwater snail *Physella acuta*as the study system [9,10]. No effect was found for vinclozolin concentrations up to 0.1 milligrams per liter (mg/L) [9], yet DNA demethylation was detected at concentrations of 5 mg/L [10]. The impact of vinclozolin and other chemicals on epigenetics in this specific gastropod and other species of this taxonomic class is still not clear.

In vertebrate cells and tissues, cadmium can induce both global hyper- and hypomethylation depending on exposure concentration and duration [22–28]. There is, however, little information linking Cd exposure to changes in global 5mC levels in invertebrates [29], and especially land snails, although they serve as pertinent bioindicators for environmental cadmium. The temporal dynamics of contaminant effects on different epigenetic marks in invertebrates also remains to be determined. Moreover, the usefulness of 5mC-based signatures triggered by trace metals in ecotoxicology is poorly understood. Therefore, we evaluated, for the first time, the global 5mC levels in DNA isolated from the hepatopancreas of land snails with the aim to determine whether this epigenetic mark is responsive to Cd exposure. We implemented a continuous exposure, multiple dose- and time-point study design (14, 28, and 56 days) and used *Cantareus aspersus* as the study system. Cadmium levels in the hepatopancreas were determined using Flame Atomic Absorption Spectrometry and the percentage of 5-mC in samples with an ELISA-based colorimetric assay. Snail death rates were used as a lethality endpoint. Statistical analysis was then used to estimate when and to which extent Cd exposure modifies the global 5mC content of genomic DNA in hepatopancreas tissue. Using epigenetic approaches to address environmental problems is an emerging trend in ecotoxicology [30] and constitutes one of the next frontiers in the study of epigenetics [31].

## Results

It has been demonstrated that terrestrial gastropods can be successfully reared under laboratory conditions using agar-based foods [13,32]. Similarly, the food used throughout the present experiment was well accepted by the snails. The average concentrations of Cd in the food were (i) for the 0Cd treatment, below the detection limit (0.01 mg/kg d. wt); (ii) for the 0.05Cd treatment, 0.05 ± 0.02 mg/kg d. wt; (ii) for the 0.2Cd treatment, 0.19 ± 0.04 mg/kg d. wt; (iv) for the 1Cd treatment,0.99 ± 0.17mg/kg d. wt; (v) for the 10Cd treatment, 10.58 ± 1.29 mg/kg d. wt; (vi) for the 100Cd treatment, 99.79 ± 6.55 mg/kg d. wt. The consumption rates were not measured, but 50 days after the start of the experiment 4 specimens (8.16%) for the 100Cd treatment and 7 specimens (5.76%) for the 10Cd treatment, respectively, were found to avoid food and then become dormant.

### Hepatopancreas cadmium concentrations

**Fig 1:**
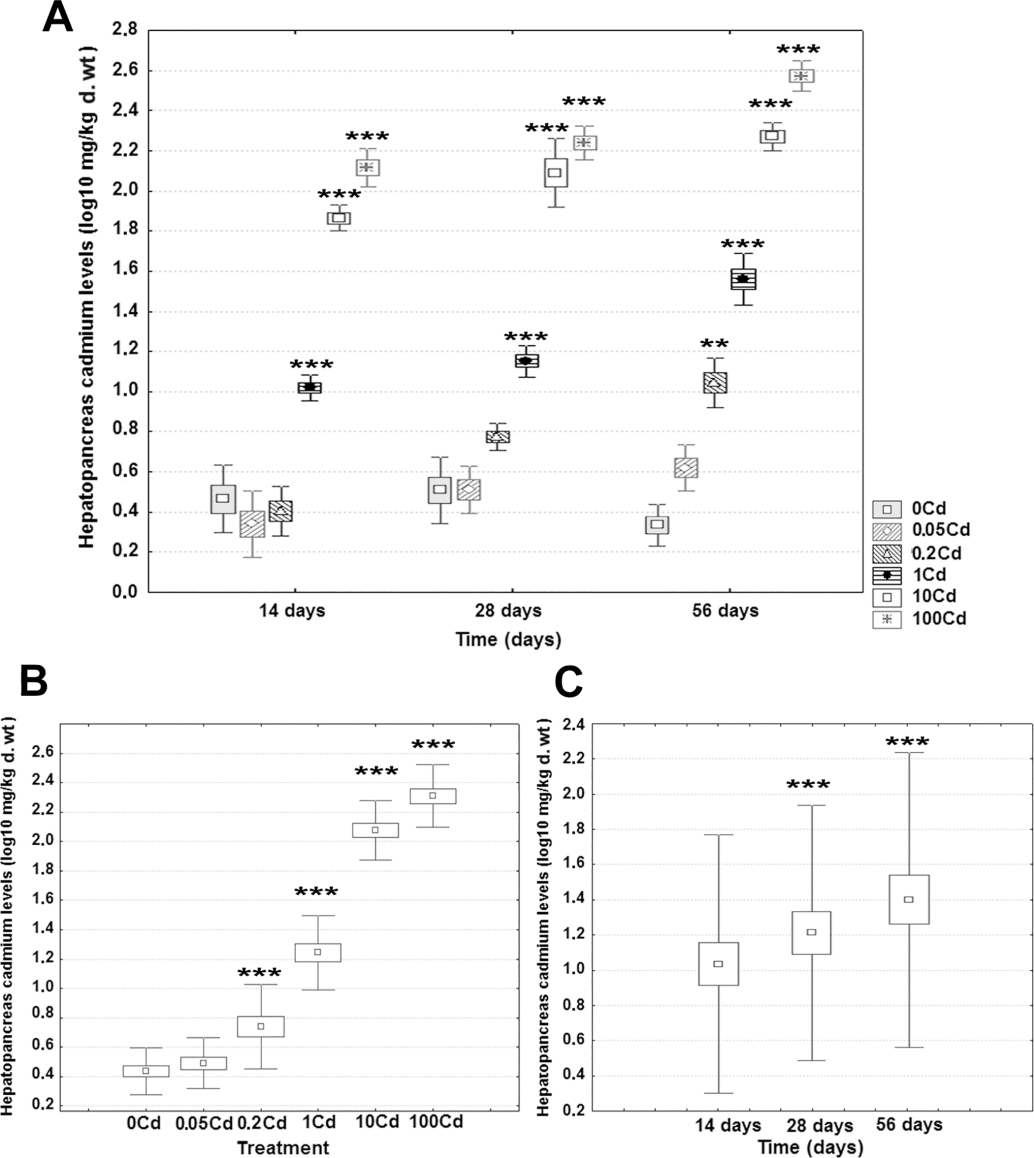
Effect of dietary exposure on cadmium retention in the hepatopancreas of *C*. *aspersus* adults. (**A**) Average cadmium concentrations for each treatment group at each time point. (**B**) Estimated marginal means for dietary dose (as the main effect). (**C**) Estimated marginal means for time (as the main effect). Six snails were sampled for each treatment group at each time point (14, 28, and 56 days). Data are shown on a log_10_ scale as mean (point) with one standard error (box) and one standard deviation (error bar). In Fig 1A the outliers (white circles) and the extremes (whiskers) are also given (if they exist). Marked boxes(*) indicate significant differences as compared to the reference group (Newman-Keuls test, ***—*p* < 0.001, **—*p* < 0.01, *—*p* < 0.05).

Average concentrations of Cd in the hepatopancreas of controls were between 2.23 and 3.43mg/kg d. wt, whereas in exposed animals they ranged from 2.31 mg/kg d. wt in the 0.05Cd snails at 14 days up to 377.63 mg/kg d. wt in the 100Cd exposed snails at 56 days (Fig 1A). Detailed information about cadmium levels in the hepatopancreas of all specimens sampled is available in the S1 Table. The log_10_-transformed data for Cd levels and 5mC content of the hepatopancreas were normally distributed (Anderson-Darling test, *p* ≥ 0.062). The tests for homogeneity of variancewere not significant (Levene's test, *p* ≥ 0.057), indicating that this assumption underlying the application of the two-way ANOVA was met for both variables. A significant effect of dietary dose on Cd retention in the hepatopancreas was observed (F(5, 102) = 876.75, *p* <0.001, η^2^ = 0.923). Post hoc analyses using the Newman-Keuls procedure indicated a significant dose-dependent increase in the measured values from the second lowest dose onward (Fig 1B).

We also found that the main effect of exposure duration on hepatopancreas cadmium was significant (F(2, 105) = 88.83, *p* < 0.001, η^2^ = 0.037). However, the relative impact of this variable was more than 30-fold lower as compared to that of Cd dose. There was a significant increase in hepatopancreas Cd concentrations during the 15–56 days period, as shown by post hoc analyses using Newman-Keuls tests (Fig 1C). The meaning of this effect was qualified, however, by a significant outcome of the interaction between dietary dose x exposure duration (F(10, 97) = 9.53, *p* < 0.001, η^2^ = 0.021). That is, the main effect of exposure duration depends on the level of dietary cadmium. At 14 days of exposure (Fig 1A),Cd concentrations were significantly higher in the 1Cd, 10Cd, and 100Cd snails compared to the unexposed controls, but not for the two lowest treatments. At 28 days (Fig 1A), a substantive increase in hepatopancreas cadmium was observed for most treatment groups, with the exception of specimens fed on the lowest dietary dose. This trend was even more noticeable at 56 days (Fig 1A), when all treatments were associated with significantly enhanced Cd levels in the hepatopancreas of *C*. *aspersus* relative to the reference group.

### Genome-wide DNA methylation in the hepatopancreas

**Fig 2:**
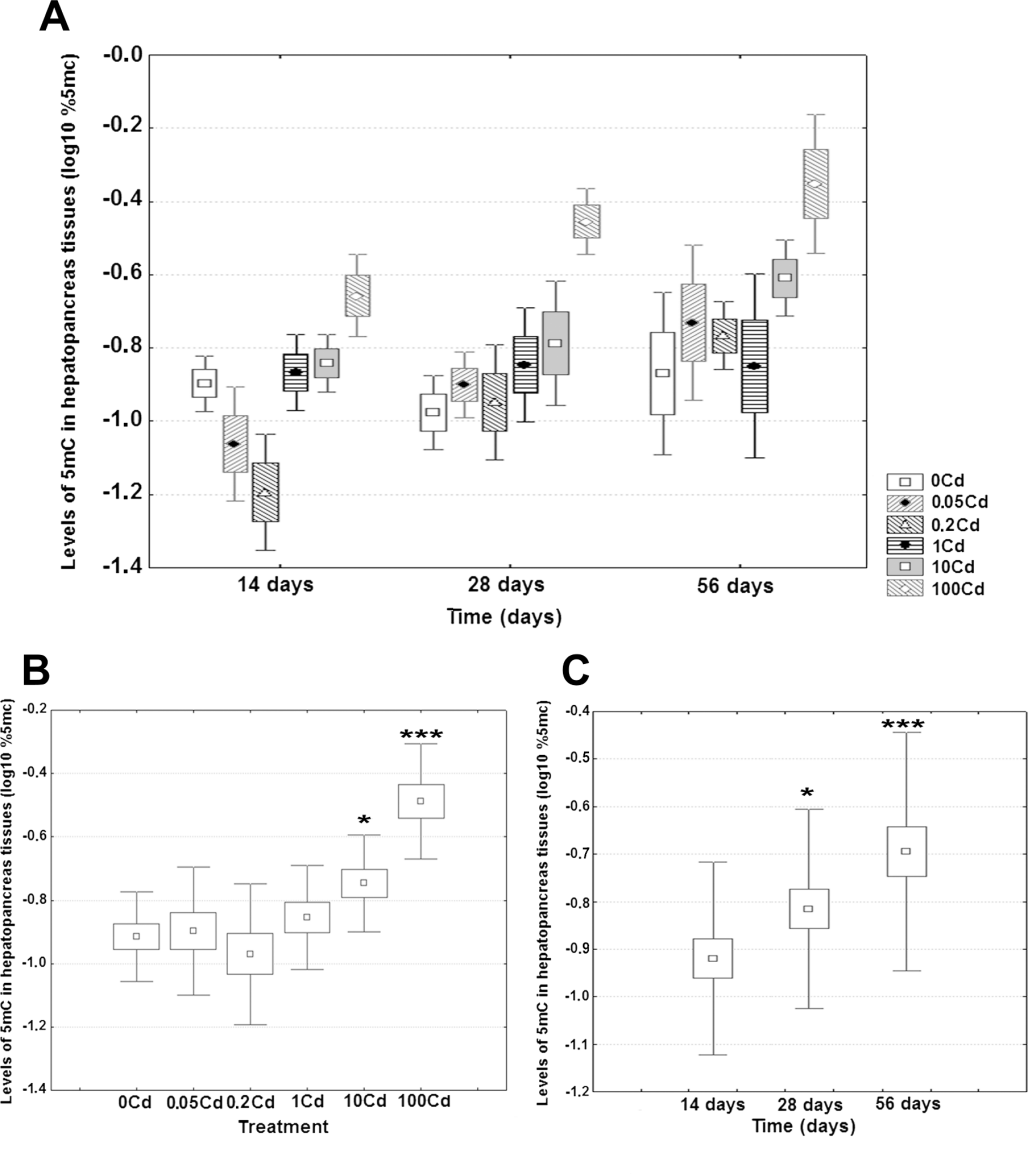
Effect of dietary cadmium on genome-wide 5mC levels in DNA of the hepatopancreas of *C*. *aspersus* adults. (**A**) The mean contents of 5mC in hepatopancreas DNA in each treatment group at each time point. (**B**) Estimated marginal means for dietary dose (as the main effect). (**C**) Estimated marginal means for time (as the main effect). Four snails were sampled for each treatment group at each time point (14, 28, and 56 days). Data are shown on a log_10_ scale as mean (point) with one standard error (box) and one standard deviation (error bar). In Fig 2A the outliers (white circles) and the extremes (whiskers) are also given (if they exist). Marked boxes(*) indicate significant differences as compared to the reference group (Newman-Keuls test, ***—*p* < 0.001, **—*p* < 0.01, *—*p* < 0.05).

Average levels of 5mC in the hepatopancreas ranged between 0.067% in the 0.2Cd snails at 14 days and 0.479% in the 100Cd snails at 56 days, with an overall weighted mean for all groups of 0.176% (Fig 2A). Detailed data about genome-wide methylation levels in DNA extracted from the snail hepatopancreas are given in the S2 Table. There was a significant main effect of dietary dose on global DNA methylation of the hepatopancreas (F(5, 66) = 16.55, *p* < 0.001, η^2^ = 0.464). Application of post-hoc pairwise comparisons using the Newman-Keuls method revealed a significant elevation in 5mC levels from the second highest dose onward (Fig 2B).

The main effect of exposure duration on total 5mC level of the hepatopancreas was significant (F(2, 69) = 13.47, *p* < 0.001, η^2^ = 0.151), but the relative impact of this variable was more than three-fold weaker compared to dietary dose. When compared to the first time point, the measured values increased significantly with time during the 15–28 days period (Fig 2C). The interaction dietary dose x exposure duration was not statistically significant (F(10, 61) = 1.44, *p* = 0.184, η^2^ = 0.083). However, a strong association was observed between hepatopancreas cadmium levels and the corresponding 5mC levels (*r* = 0.63, *p* < 0.001; for details on paired data sets see the S2 Table).

### Cadmium lethality

The lowest average survival times and the highest death rates tended to occur for the two highest treatments, irrespective of time point (Table 1). Mortalities ranged between 4.62% in the 0.05Cd snails for the 15–28 days period and 12.24% in the 100Cd snails for the 29–56 days period (Table 1).There was no consistent difference in death ratesamong controls (Chi-Square test, *p* = 0.457). We also found no significant differences in survival times across groupsat 14 days (Log-Rank test, *p* = 0.907). Identical results were obtained at 28 days (Log-Rank test, *p* = 0.796) and 56 days (Log-Rank test, *p* = 0.762). Detailed information about the survival time for snails in each group and the corresponding data type (censored vs. complete) are shown in the S3 Table.

**Table 1:** Survival times and mortalities for each time period.

	Period 0–14 days				Period 15–28 days				Period 29–56 days			
Treatment	Survival time		Mortalities		Survival time		Mortalities		Survival time		Mortalities	
100Cd	13,32	(2,49)	9.33%	(7)	27,44	(1,74)	11.29%	(7)	54,18	(5,86)	12.24%	(6)
10Cd	13,64	(1,51)	8.00%	(6)	27,59	(1,50)	7.94%	(5)	55,06	(4,03)	7.69%	(4)
1Cd	13,69	(1,50)	8.00%	(6)	27,30	(2,52)	7.94%	(5)	55,62	(5,41)	5.74%	(3)
0.2Cd	13,72	(1,52)	6.67%	(5)	27,63	(1,85)	6.25%	(4)	55,15	(4,08)	7.41%	(4)
0.05Cd	13,72	(1,36)	5.33%	(4)	27,66	(1,71)	4.62%	(3)	54,95	(4,50)	10.71%	(6)
0.0Cd	13,75	(1,43)	6.67%	(5)	27,78	(1,09)	6.25%	(4)	54,74	(4,43)	9.26%	(5)

The survival times for each treatment were measured at the end of each time period as the area under the Kaplan-Meier estimate of the corresponding survival curve and are expressed as mean values with one standard error (in paranthesis). The mortalities are shown in percentages (the number of dead snails at the end of each time period in paranthesis).

## Discussion

### Hepatopancreas cadmium concentrations

Land snails are considered to be 'macroconcentrator' species for Cd based on their exceptional ability to accumulate it in their tissues at levels which by far exceed those encountered in their surrounding environment[33]. Several studies have examined the retention of dietary Cd in the soft tissues of *C*. *aspersus* under laboratory conditions, but not at environmentally-relevant exposure levels below 0.2 mg/kg d. wt [13,34,35].The very low cadmium concentrations measured repeatedly in controls at different time points demonstrate that ingestion of food was the only exposure path observed and confirm that the experiment was properly conducted. The values of control animals are close to those found in adult *C*. *aspersus* originating from relatively unpolluted areas, with normal levels of Cd in soils and/or vegetation [36,37]. That is, small amounts of cadmium, such as those reported here for controls, are unlikely the result of exposure to a contaminated environment prior to the start of this experiment; rather these are Cd levels normally encountered in the hepatopancreas of *C*. *aspersus* snails.

The measured values in exposed animals showed a tendency to increase with increasing dose and duration, with the former variable being the most influential factor. This increasing trend in Cd accumulation was particularly evident for the three highest treatments.Our findings are consistent with an earlier laboratory study, which found marked elevation of Cd levels in the soft tissues of *C*. *aspersus* of comparable size for exposures above 1.11 ± 0.07 mg/kg d. wt in the food [13]. The present results also indicate that prolonged exposure to dietary doses as low as 0.2 mg/kg d. wt Cd as cadmium chloride can result in significant elevation of Cd levels in the hepatopancreas of mature snails, *C*. *aspersus*. Moreover, the dynamics of metal retention observed in the two lowest treatment groups suggests that this accumulation only occurs if the hepatopancreas Cdlevels surpassa certain threshold level in gastropods; under the present experimental conditions this would be around 12 mg/kg d. wt.

The maximum cadmium contents observed in this study were above 400 mg/kg d. wt, and hence among the greatest values reported in *C*. *aspersus* for exposure via the diet. Similar results were reported in (eco)toxicological studies investigating the retention of dietary Cd in other terrestrial gastropods, such as *Arianta arbustorum* (Linnaeus, 1758) [38], *Cepaea hortensis* (Müller, 1774) [39], or *Cantareus apertus* (Born, 1778) [40]. Because the measured values did not reach equilibrium, it is likely that this species can also accumulate cadmium in the hepatopancreas at higher levels than those seen here. In fact, such elevated concentrations have already been documented for other species of land snails. Thus, *Cepaea hortensis* retained Cd in the hepatopancreas at levels above 1,600 mg/kg d. wt following 14 days exposure to up to 250 mg/kg d. wt in the food [41].

### Genome-wide DNA methylation in the hepatopancreas

This is the first study to document the presence of 5mC in *C*. *aspersus*. By using a hepatopancreas-targeted approach, this paper also significantly expands previous knowledge on DNA methylation in gastropods, which until now was limited to data derived from whole-body [8–10], soft tissue of the foot [7], or neurons [42]. The genome-wide 5mC levels in DNA of the hepatopancreas of *C*. *aspersus* were close to those found using the same method (colorimetric-based ELISA assay) in mature specimens of *Physella acuta* and *Zeacumantus subcarinatus*, that is*~* 0.2–0.9% [8,9]. However, liquid chromatography-tandem mass spectrometry (LC-MS/MS) analysis revealed that approximately 2% of cytosine nucleotides are methylated in *Biomphalaria glabrata* [7]. These findings may result from different methodological approaches [43], as well as from tissue and species-specific differences in genome-wide DNA methylation levels [44,45].

Aging is generally accompanied by a gradual loss of genome-wide DNA methylation in eukaryotic cells and tissues [46,47]. A recent study found a consistent decrease of global 5mC content in adult *P*. *acuta* snails for an age difference of 42–70 days [9]. In contrast, in our experiment the measured values in controls stayed relatively constant during the 56 days of exposure. It is known that the former species is a short-lived gastropod, with an average life expectancy of only seven to twelve months [48], whereas *C*. *aspersus* has a mean life span in natural environments between three and six years [49,50].

Therefore, we hypothesize the existence of an inverse relationship in gastropods between a species’ longevity and age-dependent loss of DNA methylation. Indeed, such an association does exist in mammals, e.g., humans vs.mice [51], and may reflect the ability of long-lived organisms to maintain their DNA methylation during adult life more effectively than their short-lived counterparts.

### Cadmium effect onhepatopancreas DNA methylation

Here, we provide the first pertinent evidence that Cd exposure can modify global 5mC levels in DNA of gastropods and mollusks.This is consistent with the findings of previous studies that were conducted on humans, rodents, chickens, fish, and earthworms [22,23,26,27,29]. Most of these studies took into account a sublethal exposure scenario with a duration between one and three months, but none of them have used experimental designs with three or more time points to determine the temporal effects of cadmium on genome-wide DNA methylation levels; this is another key strength of the present paper.

Cadmium effect on global DNA methylation levels in the hepatopancreas of adult *C*. *aspersus* was both dose-dependent and time-dependent, with exposure dose having a more important influence than duration. We found a significant increasing trend in 5mC levels starting at 28 days of exposure to the second highest dose and this trend continued at the two highest treatments for close to one month, when the experiment was terminated after 56 days. Together with the strong correlation observed here between cadmium levels and 5mC levels in the hepatopancreas, these data indicate an overall trend towards DNA hypermethylation with elevated Cd exposure. A similar association has only very recently been reported in invertebrates, more precisely in earthworms [29], and is in line with the results derived from human studies [24,28]. Since DNA methylation is commonly associated with gene silencing [5], elevated methylation levels observed here at different time points may suggest an increased likelihood of altered gene expression in response to Cd-exposure. However, the biological significance cannot be established at this stage without further experimental evidence at transcriptional and/or translational level.

We note that mature snails, *C*. *aspersus*, concentrated substantive amounts of cadmium in the hepatopancreas without significant lethal effects. This is in accordance with the results of other investigations [13,34], confirming the exceptional tolerance of this gastropod species to Cd exposure [13,16,34,35,52,53]. Most studies to date found no significant mortality in *C*. *aspersus* adults exposed to maximum dietary doses of 100 mg/kg d. wt Cd for up to four months, but identified a dose-dependent decrease in food consumption/body weight [13,34,52,53], followed by feeding cessation and entrance to dormancy as exposure dose increased [34]. A similar trend was observed towards the end of the present experiment for a small proportion of snails given the two highest cadmium levels in the food. However, we cannot exclude that a longer duration or a higher exposure dose may lead to lethal outcomes. Thus, it was found that dietary cadmium (as cadmium chloride) induces high mortality in *C*. *aspersus* adults when given at concentrations of 200 and 400 mg/kg d. wt Cd for ten weeks [53].

Based on the present results, cytosine methylation appears to be a less sensitive endpoint of cadmium exposure when compared to hepatopancreas cadmium level. However, we were able to detect a significant, persistent hypermethylation of hepatopancreas DNA in response to sublethal doses of cadmium. This opens new paths for future studies on the implications of epigenetics in molluskan ecotoxicology. For example, genome-wide DNA methylation may not be the most important factor in epigenetic regulation, but rather, different type of DNA modification marks may form patterns of epigenetic signatures which could be used as biomarkers. It will also be of interest to study the effects of cadmium on specific genes in gastropods. In this context, the Cd-selective metallothionein (*Cd-MT*) gene deserves particular consideration since it can be activated by dietary Cd levels as low as 2 mg/L [54,55] and is generally inducible, unlike constitutively expressed genes with housekeeping functions [56]. The *Cd-MT* gene contains CG sites [57], but there is no information available about their methylation status. In the common earthworm, *Lumbricus terrestris* (Linnaeus, 1758), a very recent study found no methylationin CG pairs of a selected promotor region of the Cd-inducible metallothionein gene *wMT-2* even in the presence of cadmium exposure [58].However, the authors did not investigate the gene body region, which cannot eliminate a role of DNA methylation in the regulation of the *wMT-2* gene or a potential effect of Cd on the methylation status of CG islands in the body of this gene. Such gene-based biomarkers may provide a reliable approach for detection of subtle effects of low-level Cd exposure, as has been already described in vertebrates [25–28,59].

The main limitation of this study is the moderate within-group variation in global 5mC levels, which can decrease statistical power, and thus the chance of detecting small, but significant differences between groups. This may result from the modest sample size collected per each treatment group, which is not unusual for global DNA methylation studies due to the cost constraint. However, a similar amount of variability in total 5mC content was observed in *P*. *acuta* snails, irrespective of age and VZ dose used [9]. As a result, such variation may also reflect inter-individual differences related to the cumulated effect of multiple factors, including feeding status [60], health condition [61], and parental origin [62].

Moreover, *C*. *aspersus* and most terrestrial gastropods have yet to have their genomes sequenced. There is also surprisingly little information regarding the gastropod methylome. Further studies are hence required not only to expand on the ecotoxicological significance of our findings, but also to refine our knowledge of how the methylome regulates gene expression in terrestrial gastropods. These studies need to be performed with a larger sample size and must be designed to provide us with genome-wide and gene-specific high-resolution mapping and functional analysis of 5mC and other cytosine modification variants on individuals and different species of land snails, with emphasis on cell type, ontogenetic stage, and environmental status. Such fundamental work is a sine qua noncondition to fully understand the extent to which Cd affects the methylome of the gastropod hepatopancreas and the potential of land snails to serve as suitable bioindicators of epigenetic effects of chemicals.

## Materials and methods

No ethical approval was required for using *Cantareus aspersus* as invertebrate model in (eco)toxicological studies. However, our experiments were carried out in accordance with the internal guidelines of “Victor Babes” University of Medicine and Pharmacy from Timisoara (UMFT), Romania. These guidelines are consistent with the national and European recommendations regarding the protection and welfare of laboratory animals. The purchase of C. *aspersus* snails for this study did not involve endangered or protected species.

### Experimental design

The present work was conducted under controlled environmental conditions at the Laboratory Animal Facility of “Victor Babes” University of Medicine and Pharmacy from Timisoara (UMFT). It involved a continuous exposure design and a random, high sampling methodology, that is at 14, 28, and 56days. Similar timepoints are routinely used in studies on cadmium toxicity in humans and laboratory animals, including land snails [3,16,39,52,62].

To provide relevant results for both environmental hazard and animal exposure, the test snails were fed a Cd-enriched diet. This exposure route is the main path of Cd uptake not only in terrestrial gastropods [26], but also in humans, mammals, and most terrestrial invertebrates [3]. In concordance with previous ecotoxicological studies with terrestrial gastropods, cadmium chloride (CdCl_2,_ 99.99% trace metal basis, Sigma-Aldrich) was used as a source of cadmium [14,16,32,63]. Exposure doses covered a wide range of Cd concentrations to improve the probability of observing the impact of this non-essential trace metal on global 5mC levels in the hepatopancreas. The seven nominal Cd treatments were 0, 0.05, 0.2, 1, 10, and 100 mg/L (abbreviated as 0Cd, 0.05Cd,0.2Cd, 1Cd, 10Cd, and 100Cd). These concentrations were chosen based on the reference values for Cd effects on key toxicological endpoints: cell proliferation, histopathological alterations, and DNA integrity in the hepatopancreas of gastropods [31,64]; and maximum Cd level allowed in vegetal foods on which the snails regularly feed, such as leafy vegetables or fruits [65,66].

### Rearing conditions

Four weeks before the start of the experiment (early December 2015), 600 newly matured specimens of brown garden snail (*Cantareusaspersus* Müller 1774), aged 12 months, were purchased from the “Mokry Dwór” snail farm (Krzymów, Wielkopolska, Poland). At this life stage, the shell stops growing and develops a reinforcing lip at aperture, which serves as a sign of sexual maturity [67]. After being transferred to the laboratory, the animals were kept in groups of 25 in 30-liter aerated polypropylene containers/terrariums (70.5 x 39.5 x 18.5 cm) with perforated side walls at 18 to 20°C, under a constant light-dark cycle of 12:12 hours. The bottom section of the rearing containers was covered with a layer of ash-free filter paper (70.5 x 39.5 cm). To provide a moist microenvironment inside the containers, this layer was wetted two times a day with double distilled water by using a pressure sprayer.

During this pre-exposure period, the snails were fed an artificial food prepared by mixing 50 g fortified infant cereals (Nestle Nestum 5 –Five Cereals), 20 g carrot baby food (HiPP, UK), and 15 g agar (A-1296, Sigma) with double distilled water to yield 1000 mL agar medium. A 1% methylparaben solution (3 mL) was added to extend the storage period of the fodder (Itziou et al., 2011). Each liter of medium was distributed equally among forty 7-cm-diameter Petri dishes (about 25 mL/dish), which, after cooling, were kept in the refrigerator for maximum one week. The daily activity schedule consisted of fresh fodder supply (two Petri dishes per each terrarium), monitoring snail fitness, removing faeces and uneaten food, replacing the sheet of ash-free filter paper, cleaning the containers with sterile paper towels, and collecting the dead specimens. The rearing containers were cleaned three times a week with double distilled water.

At the beginning of the experiment (January 2016), the snails were sorted based on their size to obtain homogeneous experimental groups. To this end, both the shell height and snail weight were used to estimate the animal size. Shell height was assessed with a digital caliper to the nearest 0.01 mm; and the weight by using an analytical balance to the nearest 0.01 mg. The measurement methods were compiled from the malacological literature [63]. The mean shell size of selected snails (i.e., 450 specimens) was 2.73 ± 0.29 cm length, whereas the average weight was 8.44 ± 1.23 g.

At 14, 28, and 56 days, six snails (i.e., two specimens from each replicate) were randomly sampled and sacrificed for each treatment group. After removing the soft body from the shell using a hemostat, the hepatopancreas was collected and washed in double distilled water. Six samples were used for performing chemical analyses, and four randomly chosen samples for extracting the DNA. All samples were dried on cellulose tissue and stored at -80°C until further analyses.

### Chemical analyses

The frozen hepatopancres samples were thawed (about 1.2–1.5 g per each sample) and oven dried at 105°C for 24 hours. The samples were next weighed to the nearest 0.01 mg with an analytical balance (TP-214, Denver Instrument GmbH) and calcinated in a muffle furnace (Nabertherm B150, Lilienthal) at 550°C for 6 hours to obtain the dry weight. The ash was dissolved by wet digestion with nitric acid, which is one of the most common methods used for analytical determination of Cd in animal samples (Faroon et al., 2012). Briefly, after being treated with 0.5 mL of HNO_3_ suprapure (Merck, 65% suprapure) and heated on a hot plate to dryness, the ash was dissolved in 20mL of 0.5N HNO_3_ and filtered through ash-free filter paper. Finally, each sample volume was brought to 30mL with 10mL HNO_3_ 0.5N.

Cadmium concentrations in the filtrates were measured by flame (air-acetylene) atomic absorption spectrometry (VARIAN AA240FS) fitted with a Cd-specific hollow-cathode lamp as a source of radiation. The results obtained were expressed as milligram per kilogram dry weight (mg/kgd. wt). Mix standard solutions (1000 mg/L) of Cd-ICP multi-element standard solution IV CertiPUR were purchased from Merck. The reagents and standard solutions were prepared using spectroscopically pure double distilled water. After being treated with a Pierce solution 20% (v/v) and rinsed with cold tap water, all glassware was washed with 20% (v/v) nitric acid and rinsed again with double distilled water. Blanks and triplicate samples were also analyzed during the procedure to provide us with homogeneous and accurate results. For quality assurance, the NCS Certified Reference Material85105a (China National Analysis Center for Iron&Steel) was used. The percent recovery was, on average, 96%, whereas the coefficients of variation fell below 8%. Cadmium quantification limit, as determined via the calibration curve, was 0.01 mg/kgd. wt. The blank reagent and standard reference animal sample were included into each sample batch to verify the accuracy and precision of the digestion procedure, as well as for the subsequent analyses. The spectrometer was recalibrated every 15 samples and blank samples were run every 25 samples.

### DNA extraction and 5mC quantification analysis

Total DNA was isolated from the hepatopancreatic tissue with the DNeasy Blood & Tissue Kit (Qiagen, Cat no. 69506) as per manufacturer’s instruction and then checked for quality (260/280 nm, NanoDrop-2000, Thermo Fisher Scientific Inc., USA). Total 5mC content in DNA of the hepatopancreas of *C*. *aspersus* was determined using an ELISA-based global DNA methylation assay. This assay was chosen because it serves as a cost-effective, fast, and reliable alternative to the routinely used methods for genome-wide mapping of DNA methylation, e.g., chromatography, radioactive filter-binding, and bisulfite sequencing-based methods [68,69], especially for serial measurements, which was the case of our study. We used the MethylFlash™ Methylated DNA Quantification Kit (EpiGentek, Cat no. P-1034-96) according to the manufacturer’s instructions. The optical density (OD) was measured at 450 nanometers (nm) using a microplate Reader Stat Fax 4200 (Awareness Technology, USA). For absolute 5-mC quantifications, a standard curve was generated by plotting the various concentrations of the positive controls against the corresponding ODs.

### Statistical analysis

Hepatopancreas cadmium was analyzed in a two-way ANOVA, with Cd dose (0Cd, 0.05Cd, 0.2Cd, 1Cd, 10Cd, 100Cd) and exposure time (14 days, 28 days, 56 days) as between subjects variables. Prior to the ANOVAtest, the log-transformed data (decimal logarithmation) were checked for normality using Anderson-Darling tests; and homogeneity of variances for each combination of the groups of the two independent variables with Levene’s tests. Posthoc analysis was performed using the Newman-Keuls approach [70]. For each significant main effect, all pairwise comparisons were made against the earliest time point. In the case of a significant interaction effect between the two predictors, these comparisons were performed at each time point using respectively the corresponding controls as benchmark groups. A similar approach was used for 5mC levels in the hepatopancreas of *C*. *aspersus* adults. Finally, a Pearson’s correlation was applied on pooled data for 5mC levels and log_10_-transformed concentrations of Cd in the hepatopancreas.

Time since initiating treatment was grouped into periods of 0–14, 15–28, and 29–56 days. Death rates of control groups at different time points were analyzed using a Chi-Square test. This aimed at eliminating the potential effect of factors other than cadmium exposure on snail mortalities, e.g., the stress induced by acclimation to a new environment. Log-Rank tests were next used for each time period to compare between snail survival across different groups. In case of significant differences among treatments, Breslow’s tests were conducted using the controls as the reference group. The proportion of the snails surviving at successive times for each treatment was assessed using a Kaplan-Meier curve. The average survival time was determined as the area under the Kaplan-Meier estimate of the survival curve. The treatment was considered complete for the dead snails and censored for the specimens who were still alive at the end of the study. Statistical significance was defined at *p* less than 0.05.

## Supporting information

S1 Table Raw data for Fig 1. (DOCX)

S2 Table Raw data for Fig 2 and for measuring the degree of correlation between Cd levels and 5mC levels in the hepatopancreas of adult *C*. *aspersus*. (DOCX)

S3 Table Raw data for generating Table 1. (DOCX)
