# Supplementary material for: High-level dietary cadmium exposure is associated with global DNA hypermethylation in the gastropod hepatopancreas
Source: PLoS One. 2017 Sep 6;12(9):e0184221. doi: 10.1371/journal.pone.0184221 (PMC5587323; doi:10.1371/journal.pone.0184221)
Supplement: S1 Table — (DOCX) [file pone.0184221.s001.docx]

| **Cd levels** | **Cd levels** | **Time** | **Treatment** |
| --- | --- | --- | --- |
| (mg kg^-1^ DW) | (log_10_ mg kg^-1^ DW) | (days) |  |
| 2.00 | 0.30 | 14 | 0Cd |
| 2.70 | 0.43 | 14 | 0Cd |
| 4.20 | 0.62 | 14 | 0Cd |
| 3.64 | 0.56 | 14 | 0Cd |
| 4.35 | 0.64 | 14 | 0Cd |
| 1.72 | 0.24 | 14 | 0Cd |
| 1.90 | 0.28 | 14 | 0.05Cd |
| 3.64 | 0.56 | 14 | 0.05Cd |
| 1.31 | 0.12 | 14 | 0.05Cd |
| 2.76 | 0.44 | 14 | 0.05Cd |
| 2.67 | 0.43 | 14 | 0.05Cd |
| 1.63 | 0.21 | 14 | 0.05Cd |
| 2.00 | 0.30 | 14 | 0.2Cd |
| 1.75 | 0.24 | 14 | 0.2Cd |
| 2.61 | 0.42 | 14 | 0.2Cd |
| 3.95 | 0.60 | 14 | 0.2Cd |
| 2.75 | 0.44 | 14 | 0.2Cd |
| 2.61 | 0.42 | 14 | 0.2Cd |
| 8.97 | 0.95 | 14 | 1Cd |
| 11.15 | 1.05 | 14 | 1Cd |
| 10.24 | 1.01 | 14 | 1Cd |
| 10.36 | 1.02 | 14 | 1Cd |
| 13.47 | 1.13 | 14 | 1Cd |
| 9.14 | 0.96 | 14 | 1Cd |
| 82.85 | 1.92 | 14 | 10Cd |
| 81.43 | 1.91 | 14 | 10Cd |
| 74.23 | 1.87 | 14 | 10Cd |
| 68.06 | 1.83 | 14 | 10Cd |
| 79.70 | 1.90 | 14 | 10Cd |
| 55.78 | 1.75 | 14 | 10Cd |
| 126.48 | 2.10 | 14 | 100Cd |
| 119.62 | 2.08 | 14 | 100Cd |
| 104.44 | 2.02 | 14 | 100Cd |
| 199.92 | 2.30 | 14 | 100Cd |
| 117.09 | 2.07 | 14 | 100Cd |
| 133.89 | 2.13 | 14 | 100Cd |
| 5.97 | 0.78 | 28 | 0Cd |
| 2.30 | 0.36 | 28 | 0Cd |
| 3.49 | 0.54 | 28 | 0Cd |
| 2.25 | 0.35 | 28 | 0Cd |
| 3.85 | 0.59 | 28 | 0Cd |
| 2.72 | 0.43 | 28 | 0Cd |
| 2.63 | 0.42 | 28 | 0.05Cd |
| 4.90 | 0.69 | 28 | 0.05Cd |
| 2.97 | 0.47 | 28 | 0.05Cd |
| 3.79 | 0.58 | 28 | 0.05Cd |
| 2.28 | 0.36 | 28 | 0.05Cd |
| 3.54 | 0.55 | 28 | 0.05Cd |
| 6.32 | 0.80 | 28 | 0.2Cd |
| 4.91 | 0.69 | 28 | 0.2Cd |
| 4.89 | 0.69 | 28 | 0.2Cd |
| 6.51 | 0.81 | 28 | 0.2Cd |
| 6.40 | 0.81 | 28 | 0.2Cd |
| 6.94 | 0.84 | 28 | 0.2Cd |
| 17.75 | 1.25 | 28 | 1Cd |
| 13.51 | 1.13 | 28 | 1Cd |
| 17.29 | 1.24 | 28 | 1Cd |
| 11.17 | 1.05 | 28 | 1Cd |
| 13.50 | 1.13 | 28 | 1Cd |
| 12.97 | 1.11 | 28 | 1Cd |
| 166.91 | 2.22 | 28 | 10Cd |
| 164.89 | 2.22 | 28 | 10Cd |
| 90.58 | 1.96 | 28 | 10Cd |
| 142.92 | 2.16 | 28 | 10Cd |
| 152.66 | 2.18 | 28 | 10Cd |
| 63.77 | 1.80 | 28 | 10Cd |
| 200.60 | 2.30 | 28 | 100Cd |
| 180.68 | 2.26 | 28 | 100Cd |
| 119.88 | 2.08 | 28 | 100Cd |
| 165.10 | 2.22 | 28 | 100Cd |
| 194.63 | 2.29 | 28 | 100Cd |
| 191.92 | 2.28 | 28 | 100Cd |
| 2.01 | 0.30 | 56 | 0Cd |
| 2.90 | 0.46 | 56 | 0Cd |
| 2.24 | 0.35 | 56 | 0Cd |
| 2.71 | 0.43 | 56 | 0Cd |
| 1.53 | 0.18 | 56 | 0Cd |
| 1.95 | 0.29 | 56 | 0Cd |
| 2.66 | 0.42 | 56 | 0.05Cd |
| 4.06 | 0.61 | 56 | 0.05Cd |
| 4.39 | 0.64 | 56 | 0.05Cd |
| 3.87 | 0.59 | 56 | 0.05Cd |
| 5.70 | 0.76 | 56 | 0.05Cd |
| 5.01 | 0.70 | 56 | 0.05Cd |
| 7.13 | 0.85 | 56 | 0.2Cd |
| 11.2 | 1.05 | 56 | 0.2Cd |
| 11.79 | 1.07 | 56 | 0.2Cd |
| 11.33 | 1.05 | 56 | 0.2Cd |
| 17.47 | 1.24 | 56 | 0.2Cd |
| 10.09 | 1.00 | 56 | 0.2Cd |
| 26.46 | 1.42 | 56 | 1Cd |
| 27.58 | 1.44 | 56 | 1Cd |
| 32.83 | 1.52 | 56 | 1Cd |
| 56.43 | 1.75 | 56 | 1Cd |
| 37.23 | 1.57 | 56 | 1Cd |
| 45.89 | 1.66 | 56 | 1Cd |
| 144.43 | 2.16 | 56 | 10Cd |
| 168.99 | 2.23 | 56 | 10Cd |
| 177.08 | 2.25 | 56 | 10Cd |
| 217.31 | 2.34 | 56 | 10Cd |
| 202.84 | 2.31 | 56 | 10Cd |
| 215.76 | 2.33 | 56 | 10Cd |
| 378.13 | 2.58 | 56 | 100Cd |
| 341.13 | 2.53 | 56 | 100Cd |
| 302.21 | 2.48 | 56 | 100Cd |
| 499.47 | 2.70 | 56 | 100Cd |
| 391.67 | 2.59 | 56 | 100Cd |
| 353.18 | 2.55 | 56 | 100Cd |
