# Supplementary material for: High-level dietary cadmium exposure is associated with global DNA hypermethylation in the gastropod hepatopancreas
Source: PLoS One. 2017 Sep 6;12(9):e0184221. doi: 10.1371/journal.pone.0184221 (PMC5587323; doi:10.1371/journal.pone.0184221)
Supplement: S2 Table — (DOCX) [file pone.0184221.s002.docx]

| **5mC levels** | **5mC levels** | **Cd levels** | **Time** | **Treatment** |
| --- | --- | --- | --- | --- |
| (%5mC) | (log_10_ %5mC) | (mg/kg d. wt) | (days) |  |
| 0.110 | -0.959 | 4.35 | 14 | 0Cd |
| 0.163 | -0.788 | 2.00 | 14 | 0Cd |
| 0.125 | -0.903 | 2.70 | 14 | 0Cd |
| 0.116 | -0.936 | 4.20 | 14 | 0Cd |
| 0.141 | -0.851 | 3.64 | 14 | 0.05Cd |
| 0.063 | -1.201 | 2.76 | 14 | 0.05Cd |
| 0.070 | -1.155 | 1.31 | 14 | 0.05Cd |
| 0.091 | -1.041 | 1.63 | 14 | 0.05Cd |
| 0.061 | -1.215 | 2.00 | 14 | 0.2Cd |
| 0.039 | -1.409 | 2.75 | 14 | 0.2Cd |
| 0.081 | -1.092 | 3.95 | 14 | 0.2Cd |
| 0.087 | -1.060 | 2.61 | 14 | 0.2Cd |
| 0.134 | -0.873 | 8.97 | 14 | 1Cd |
| 0.110 | -0.959 | 11.15 | 14 | 1Cd |
| 0.122 | -0.914 | 10.24 | 14 | 1Cd |
| 0.190 | -0.721 | 9.14 | 14 | 1Cd |
| 0.158 | -0.801 | 82.85 | 14 | 10Cd |
| 0.159 | -0.799 | 74.23 | 14 | 10Cd |
| 0.110 | -0.959 | 68.06 | 14 | 10Cd |
| 0.155 | -0.810 | 55.78 | 14 | 10Cd |
| 0.189 | -0.724 | 126.48 | 14 | 100Cd |
| 0.213 | -0.672 | 119.62 | 14 | 100Cd |
| 0.321 | -0.493 | 104.44 | 14 | 100Cd |
| 0.184 | -0.735 | 117.09 | 14 | 100Cd |
| 0.131 | -0.883 | 2.30 | 28 | 0Cd |
| 0.116 | -0.936 | 3.85 | 28 | 0Cd |
| 0.076 | -1.119 | 3.49 | 28 | 0Cd |
| 0.107 | -0.971 | 2.72 | 28 | 0Cd |
| 0.103 | -0.987 | 2.63 | 28 | 0.05Cd |
| 0.146 | -0.836 | 2.97 | 28 | 0.05Cd |
| 0.108 | -0.967 | 3.79 | 28 | 0.05Cd |
| 0.154 | -0.812 | 2.28 | 28 | 0.05Cd |
| 0.067 | -1.174 | 6.32 | 28 | 0.2Cd |
| 0.118 | -0.928 | 4.90 | 28 | 0.2Cd |
| 0.135 | -0.870 | 4.89 | 28 | 0.2Cd |
| 0.152 | -0.818 | 6.94 | 28 | 0.2Cd |
| 0.158 | -0.801 | 17.29 | 28 | 1Cd |
| 0.227 | -0.644 | 11.17 | 28 | 1Cd |
| 0.105 | -0.979 | 13.50 | 28 | 1Cd |
| 0.11 | -0.959 | 12.97 | 28 | 1Cd |
| 0.278 | -0.556 | 166.91 | 28 | 10Cd |
| 0.117 | -0.932 | 120.22 | 28 | 10Cd |
| 0.128 | -0.893 | 90.58 | 28 | 10Cd |
| 0.171 | -0.767 | 142.92 | 28 | 10Cd |
| 0.369 | -0.433 | 200.60 | 28 | 100Cd |
| 0.278 | -0.556 | 180.68 | 28 | 100Cd |
| 0.453 | -0.344 | 119.88 | 28 | 100Cd |
| 0.326 | -0.487 | 191.92 | 28 | 100Cd |
| 0.188 | -0.726 | 2.01 | 28 | 0Cd |
| 0.147 | -0.833 | 2.90 | 56 | 0Cd |
| 0.064 | -1.194 | 1.53 | 56 | 0Cd |
| 0.189 | -0.724 | 1.95 | 56 | 0Cd |
| 0.120 | -0.921 | 4.06 | 56 | 0.05Cd |
| 0.134 | -0.873 | 3.87 | 56 | 0.05Cd |
| 0.215 | -0.668 | 4.39 | 56 | 0.05Cd |
| 0.346 | -0.461 | 5.70 | 56 | 0.05Cd |
| 0.158 | -0.801 | 7.13 | 56 | 0.2Cd |
| 0.183 | -0.738 | 11.20 | 56 | 0.2Cd |
| 0.134 | -0.873 | 11.79 | 56 | 0.2Cd |
| 0.222 | -0.654 | 17.47 | 56 | 0.2Cd |
| 0.285 | -0.545 | 26.46 | 56 | 1Cd |
| 0.138 | -0.860 | 27.58 | 56 | 1Cd |
| 0.148 | -0.830 | 32.83 | 56 | 1Cd |
| 0.069 | -1.161 | 56.43 | 56 | 1Cd |
| 0.263 | -0.580 | 144.43 | 56 | 10Cd |
| 0.237 | -0.625 | 168.99 | 56 | 10Cd |
| 0.182 | -0.740 | 177.08 | 56 | 10Cd |
| 0.322 | -0.492 | 217.31 | 56 | 10Cd |
| 0.299 | -0.524 | 378.13 | 56 | 100Cd |
| 0.489 | -0.311 | 341.13 | 56 | 100Cd |
| 0.337 | -0.472 | 391.67 | 56 | 100Cd |
| 0.792 | -0.101 | 353.18 | 56 | 100Cd |
