# Supplementary material for: High-level dietary cadmium exposure is associated with global DNA hypermethylation in the gastropod hepatopancreas
Source: PLoS One. 2017 Sep 6;12(9):e0184221. doi: 10.1371/journal.pone.0184221 (PMC5587323; doi:10.1371/journal.pone.0184221)
Supplement: S3 Table — (DOCX) [file pone.0184221.s003.docx]

| **Treatment /** | **Specimens** | **Survival time** | **Data type** |
| --- | --- | --- | --- |
| **exposure duration** | **(number)** | **(days)** |  |
| 100Cd, 14d | 1 | 2 | complete |
| 100Cd, 14d | 2 | 2 | complete |
| 100Cd, 14d | 3 | 3 | complete |
| 100Cd, 14d | 4 | 7 | complete |
| 100Cd, 14d | 5 | 9 | complete |
| 100Cd, 14d | 6 | 11 | complete |
| 100Cd, 14d | 7 | 13 | complete |
| 100Cd, 14d | 8 | 14 | censored |
| 100Cd, 14d | 9 | 14 | censored |
| 100Cd, 14d | 10 | 14 | censored |
| 100Cd, 14d | 11 | 14 | censored |
| 100Cd, 14d | 12 | 14 | censored |
| 100Cd, 14d | 13 | 14 | censored |
| 100Cd, 14d | 14 | 14 | censored |
| 100Cd, 14d | 15 | 14 | censored |
| 100Cd, 14d | 16 | 14 | censored |
| 100Cd, 14d | 17 | 14 | censored |
| 100Cd, 14d | 18 | 14 | censored |
| 100Cd, 14d | 19 | 14 | censored |
| 100Cd, 14d | 20 | 14 | censored |
| 100Cd, 14d | 21 | 14 | censored |
| 100Cd, 14d | 22 | 14 | censored |
| 100Cd, 14d | 23 | 14 | censored |
| 100Cd, 14d | 24 | 14 | censored |
| 100Cd, 14d | 25 | 14 | censored |
| 100Cd, 14d | 26 | 14 | censored |
| 100Cd, 14d | 27 | 14 | censored |
| 100Cd, 14d | 28 | 14 | censored |
| 100Cd, 14d | 29 | 14 | censored |
| 100Cd, 14d | 30 | 14 | censored |
| 100Cd, 14d | 31 | 14 | censored |
| 100Cd, 14d | 32 | 14 | censored |
| 100Cd, 14d | 33 | 14 | censored |
| 100Cd, 14d | 34 | 14 | censored |
| 100Cd, 14d | 35 | 14 | censored |
| 100Cd, 14d | 36 | 14 | censored |
| 100Cd, 14d | 37 | 14 | censored |
| 100Cd, 14d | 38 | 14 | censored |
| 100Cd, 14d | 39 | 14 | censored |
| 100Cd, 14d | 40 | 14 | censored |
| 100Cd, 14d | 41 | 14 | censored |
| 100Cd, 14d | 42 | 14 | censored |
| 100Cd, 14d | 43 | 14 | censored |
| 100Cd, 14d | 44 | 14 | censored |
| 100Cd, 14d | 45 | 14 | censored |
| 100Cd, 14d | 46 | 14 | censored |
| 100Cd, 14d | 47 | 14 | censored |
| 100Cd, 14d | 48 | 14 | censored |
| 100Cd, 14d | 49 | 14 | censored |
| 100Cd, 14d | 50 | 14 | censored |
| 100Cd, 14d | 51 | 14 | censored |
| 100Cd, 14d | 52 | 14 | censored |
| 100Cd, 14d | 53 | 14 | censored |
| 100Cd, 14d | 54 | 14 | censored |
| 100Cd, 14d | 55 | 14 | censored |
| 100Cd, 14d | 56 | 14 | censored |
| 100Cd, 14d | 57 | 14 | censored |
| 100Cd, 14d | 58 | 14 | censored |
| 100Cd, 14d | 59 | 14 | censored |
| 100Cd, 14d | 60 | 14 | censored |
| 100Cd, 14d | 61 | 14 | censored |
| 100Cd, 14d | 62 | 14 | censored |
| 100Cd, 14d | 63 | 14 | censored |
| 100Cd, 14d | 64 | 14 | censored |
| 100Cd, 14d | 65 | 14 | censored |
| 100Cd, 14d | 66 | 14 | censored |
| 100Cd, 14d | 67 | 14 | censored |
| 100Cd, 14d | 68 | 14 | censored |
| 100Cd, 14d | 69 | 14 | censored |
| 100Cd, 14d | 70 | 14 | censored |
| 100Cd, 14d | 71 | 14 | censored |
| 100Cd, 14d | 72 | 14 | censored |
| 100Cd, 14d | 73 | 14 | censored |
| 100Cd, 14d | 74 | 14 | censored |
| 100Cd, 14d | 75 | 14 | censored |
| 10Cd, 14d | 1 | 5 | complete |
| 10Cd, 14d | 2 | 6 | complete |
| 10Cd, 14d | 3 | 9 | complete |
| 10Cd, 14d | 4 | 12 | complete |
| 10Cd, 14d | 5 | 12 | complete |
| 10Cd, 14d | 6 | 13 | complete |
| 10Cd, 14d | 7 | 14 | censored |
| 10Cd, 14d | 8 | 14 | censored |
| 10Cd, 14d | 9 | 14 | censored |
| 10Cd, 14d | 10 | 14 | censored |
| 10Cd, 14d | 11 | 14 | censored |
| 10Cd, 14d | 12 | 14 | censored |
| 10Cd, 14d | 13 | 14 | censored |
| 10Cd, 14d | 14 | 14 | censored |
| 10Cd, 14d | 15 | 14 | censored |
| 10Cd, 14d | 16 | 14 | censored |
| 10Cd, 14d | 17 | 14 | censored |
| 10Cd, 14d | 18 | 14 | censored |
| 10Cd, 14d | 19 | 14 | censored |
| 10Cd, 14d | 20 | 14 | censored |
| 10Cd, 14d | 21 | 14 | censored |
| 10Cd, 14d | 22 | 14 | censored |
| 10Cd, 14d | 23 | 14 | censored |
| 10Cd, 14d | 24 | 14 | censored |
| 10Cd, 14d | 25 | 14 | censored |
| 10Cd, 14d | 26 | 14 | censored |
| 10Cd, 14d | 27 | 14 | censored |
| 10Cd, 14d | 28 | 14 | censored |
| 10Cd, 14d | 29 | 14 | censored |
| 10Cd, 14d | 30 | 14 | censored |
| 10Cd, 14d | 31 | 14 | censored |
| 10Cd, 14d | 32 | 14 | censored |
| 10Cd, 14d | 33 | 14 | censored |
| 10Cd, 14d | 34 | 14 | censored |
| 10Cd, 14d | 35 | 14 | censored |
| 10Cd, 14d | 36 | 14 | censored |
| 10Cd, 14d | 37 | 14 | censored |
| 10Cd, 14d | 38 | 14 | censored |
| 10Cd, 14d | 39 | 14 | censored |
| 10Cd, 14d | 40 | 14 | censored |
| 10Cd, 14d | 41 | 14 | censored |
| 10Cd, 14d | 42 | 14 | censored |
| 10Cd, 14d | 43 | 14 | censored |
| 10Cd, 14d | 44 | 14 | censored |
| 10Cd, 14d | 45 | 14 | censored |
| 10Cd, 14d | 46 | 14 | censored |
| 10Cd, 14d | 47 | 14 | censored |
| 10Cd, 14d | 48 | 14 | censored |
| 10Cd, 14d | 49 | 14 | censored |
| 10Cd, 14d | 50 | 14 | censored |
| 10Cd, 14d | 51 | 14 | censored |
| 10Cd, 14d | 52 | 14 | censored |
| 10Cd, 14d | 53 | 14 | censored |
| 10Cd, 14d | 54 | 14 | censored |
| 10Cd, 14d | 55 | 14 | censored |
| 10Cd, 14d | 56 | 14 | censored |
| 10Cd, 14d | 57 | 14 | censored |
| 10Cd, 14d | 58 | 14 | censored |
| 10Cd, 14d | 59 | 14 | censored |
| 10Cd, 14d | 60 | 14 | censored |
| 10Cd, 14d | 61 | 14 | censored |
| 10Cd, 14d | 62 | 14 | censored |
| 10Cd, 14d | 63 | 14 | censored |
| 10Cd, 14d | 64 | 14 | censored |
| 10Cd, 14d | 65 | 14 | censored |
| 10Cd, 14d | 66 | 14 | censored |
| 10Cd, 14d | 67 | 14 | censored |
| 10Cd, 14d | 68 | 14 | censored |
| 10Cd, 14d | 69 | 14 | censored |
| 10Cd, 14d | 70 | 14 | censored |
| 10Cd, 14d | 71 | 14 | censored |
| 10Cd, 14d | 72 | 14 | censored |
| 10Cd, 14d | 73 | 14 | censored |
| 10Cd, 14d | 74 | 14 | censored |
| 10Cd, 14d | 75 | 14 | censored |
| 1Cd, 14d | 1 | 2 | complete |
| 1Cd, 14d | 2 | 10 | complete |
| 1Cd, 14d | 3 | 12 | complete |
| 1Cd, 14d | 4 | 12 | complete |
| 1Cd, 14d | 5 | 12 | complete |
| 1Cd, 14d | 6 | 13 | complete |
| 1Cd, 14d | 7 | 14 | censored |
| 1Cd, 14d | 8 | 14 | censored |
| 1Cd, 14d | 9 | 14 | censored |
| 1Cd, 14d | 10 | 14 | censored |
| 1Cd, 14d | 11 | 14 | censored |
| 1Cd, 14d | 12 | 14 | censored |
| 1Cd, 14d | 13 | 14 | censored |
| 1Cd, 14d | 14 | 14 | censored |
| 1Cd, 14d | 15 | 14 | censored |
| 1Cd, 14d | 16 | 14 | censored |
| 1Cd, 14d | 17 | 14 | censored |
| 1Cd, 14d | 18 | 14 | censored |
| 1Cd, 14d | 19 | 14 | censored |
| 1Cd, 14d | 20 | 14 | censored |
| 1Cd, 14d | 21 | 14 | censored |
| 1Cd, 14d | 22 | 14 | censored |
| 1Cd, 14d | 23 | 14 | censored |
| 1Cd, 14d | 24 | 14 | censored |
| 1Cd, 14d | 25 | 14 | censored |
| 1Cd, 14d | 26 | 14 | censored |
| 1Cd, 14d | 27 | 14 | censored |
| 1Cd, 14d | 28 | 14 | censored |
| 1Cd, 14d | 29 | 14 | censored |
| 1Cd, 14d | 30 | 14 | censored |
| 1Cd, 14d | 31 | 14 | censored |
| 1Cd, 14d | 32 | 14 | censored |
| 1Cd, 14d | 33 | 14 | censored |
| 1Cd, 14d | 34 | 14 | censored |
| 1Cd, 14d | 35 | 14 | censored |
| 1Cd, 14d | 36 | 14 | censored |
| 1Cd, 14d | 37 | 14 | censored |
| 1Cd, 14d | 38 | 14 | censored |
| 1Cd, 14d | 39 | 14 | censored |
| 1Cd, 14d | 40 | 14 | censored |
| 1Cd, 14d | 41 | 14 | censored |
| 1Cd, 14d | 42 | 14 | censored |
| 1Cd, 14d | 43 | 14 | censored |
| 1Cd, 14d | 44 | 14 | censored |
| 1Cd, 14d | 45 | 14 | censored |
| 1Cd, 14d | 46 | 14 | censored |
| 1Cd, 14d | 47 | 14 | censored |
| 1Cd, 14d | 48 | 14 | censored |
| 1Cd, 14d | 49 | 14 | censored |
| 1Cd, 14d | 50 | 14 | censored |
| 1Cd, 14d | 51 | 14 | censored |
| 1Cd, 14d | 52 | 14 | censored |
| 1Cd, 14d | 53 | 14 | censored |
| 1Cd, 14d | 54 | 14 | censored |
| 1Cd, 14d | 55 | 14 | censored |
| 1Cd, 14d | 56 | 14 | censored |
| 1Cd, 14d | 57 | 14 | censored |
| 1Cd, 14d | 58 | 14 | censored |
| 1Cd, 14d | 59 | 14 | censored |
| 1Cd, 14d | 60 | 14 | censored |
| 1Cd, 14d | 61 | 14 | censored |
| 1Cd, 14d | 62 | 14 | censored |
| 1Cd, 14d | 63 | 14 | censored |
| 1Cd, 14d | 64 | 14 | censored |
| 1Cd, 14d | 65 | 14 | censored |
| 1Cd, 14d | 66 | 14 | censored |
| 1Cd, 14d | 67 | 14 | censored |
| 1Cd, 14d | 68 | 14 | censored |
| 1Cd, 14d | 69 | 14 | censored |
| 1Cd, 14d | 70 | 14 | censored |
| 1Cd, 14d | 71 | 14 | censored |
| 1Cd, 14d | 72 | 14 | censored |
| 1Cd, 14d | 73 | 14 | censored |
| 1Cd, 14d | 74 | 14 | censored |
| 1Cd, 14d | 75 | 14 | censored |
| 0.2Cd, 14d | 1 | 2 | complete |
| 0.2Cd, 14d | 2 | 9 | complete |
| 0.2Cd, 14d | 3 | 12 | complete |
| 0.2Cd, 14d | 4 | 12 | complete |
| 0.2Cd, 14d | 5 | 14 | complete |
| 0.2Cd, 14d | 6 | 14 | censored |
| 0.2Cd, 14d | 7 | 14 | censored |
| 0.2Cd, 14d | 8 | 14 | censored |
| 0.2Cd, 14d | 9 | 14 | censored |
| 0.2Cd, 14d | 10 | 14 | censored |
| 0.2Cd, 14d | 11 | 14 | censored |
| 0.2Cd, 14d | 12 | 14 | censored |
| 0.2Cd, 14d | 13 | 14 | censored |
| 0.2Cd, 14d | 14 | 14 | censored |
| 0.2Cd, 14d | 15 | 14 | censored |
| 0.2Cd, 14d | 16 | 14 | censored |
| 0.2Cd, 14d | 17 | 14 | censored |
| 0.2Cd, 14d | 18 | 14 | censored |
| 0.2Cd, 14d | 19 | 14 | censored |
| 0.2Cd, 14d | 20 | 14 | censored |
| 0.2Cd, 14d | 21 | 14 | censored |
| 0.2Cd, 14d | 22 | 14 | censored |
| 0.2Cd, 14d | 23 | 14 | censored |
| 0.2Cd, 14d | 24 | 14 | censored |
| 0.2Cd, 14d | 25 | 14 | censored |
| 0.2Cd, 14d | 26 | 14 | censored |
| 0.2Cd, 14d | 27 | 14 | censored |
| 0.2Cd, 14d | 28 | 14 | censored |
| 0.2Cd, 14d | 29 | 14 | censored |
| 0.2Cd, 14d | 30 | 14 | censored |
| 0.2Cd, 14d | 31 | 14 | censored |
| 0.2Cd, 14d | 32 | 14 | censored |
| 0.2Cd, 14d | 33 | 14 | censored |
| 0.2Cd, 14d | 34 | 14 | censored |
| 0.2Cd, 14d | 35 | 14 | censored |
| 0.2Cd, 14d | 36 | 14 | censored |
| 0.2Cd, 14d | 37 | 14 | censored |
| 0.2Cd, 14d | 38 | 14 | censored |
| 0.2Cd, 14d | 39 | 14 | censored |
| 0.2Cd, 14d | 40 | 14 | censored |
| 0.2Cd, 14d | 41 | 14 | censored |
| 0.2Cd, 14d | 42 | 14 | censored |
| 0.2Cd, 14d | 43 | 14 | censored |
| 0.2Cd, 14d | 44 | 14 | censored |
| 0.2Cd, 14d | 45 | 14 | censored |
| 0.2Cd, 14d | 46 | 14 | censored |
| 0.2Cd, 14d | 47 | 14 | censored |
| 0.2Cd, 14d | 48 | 14 | censored |
| 0.2Cd, 14d | 49 | 14 | censored |
| 0.2Cd, 14d | 50 | 14 | censored |
| 0.2Cd, 14d | 51 | 14 | censored |
| 0.2Cd, 14d | 52 | 14 | censored |
| 0.2Cd, 14d | 53 | 14 | censored |
| 0.2Cd, 14d | 54 | 14 | censored |
| 0.2Cd, 14d | 55 | 14 | censored |
| 0.2Cd, 14d | 56 | 14 | censored |
| 0.2Cd, 14d | 57 | 14 | censored |
| 0.2Cd, 14d | 58 | 14 | censored |
| 0.2Cd, 14d | 59 | 14 | censored |
| 0.2Cd, 14d | 60 | 14 | censored |
| 0.2Cd, 14d | 61 | 14 | censored |
| 0.2Cd, 14d | 62 | 14 | censored |
| 0.2Cd, 14d | 63 | 14 | censored |
| 0.2Cd, 14d | 64 | 14 | censored |
| 0.2Cd, 14d | 65 | 14 | censored |
| 0.2Cd, 14d | 66 | 14 | censored |
| 0.2Cd, 14d | 67 | 14 | censored |
| 0.2Cd, 14d | 68 | 14 | censored |
| 0.2Cd, 14d | 69 | 14 | censored |
| 0.2Cd, 14d | 70 | 14 | censored |
| 0.2Cd, 14d | 71 | 14 | censored |
| 0.2Cd, 14d | 72 | 14 | censored |
| 0.2Cd, 14d | 73 | 14 | censored |
| 0.2Cd, 14d | 74 | 14 | censored |
| 0.2Cd, 14d | 75 | 14 | censored |
| 0.05Cd, 14d | 1 | 5 | complete |
| 0.05Cd, 14d | 2 | 8 | complete |
| 0.05Cd, 14d | 3 | 9 | complete |
| 0.05Cd, 14d | 4 | 13 | complete |
| 0.05Cd, 14d | 5 | 14 | censored |
| 0.05Cd, 14d | 6 | 14 | censored |
| 0.05Cd, 14d | 7 | 14 | censored |
| 0.05Cd, 14d | 8 | 14 | censored |
| 0.05Cd, 14d | 9 | 14 | censored |
| 0.05Cd, 14d | 10 | 14 | censored |
| 0.05Cd, 14d | 11 | 14 | censored |
| 0.05Cd, 14d | 12 | 14 | censored |
| 0.05Cd, 14d | 13 | 14 | censored |
| 0.05Cd, 14d | 14 | 14 | censored |
| 0.05Cd, 14d | 15 | 14 | censored |
| 0.05Cd, 14d | 16 | 14 | censored |
| 0.05Cd, 14d | 17 | 14 | censored |
| 0.05Cd, 14d | 18 | 14 | censored |
| 0.05Cd, 14d | 19 | 14 | censored |
| 0.05Cd, 14d | 20 | 14 | censored |
| 0.05Cd, 14d | 21 | 14 | censored |
| 0.05Cd, 14d | 22 | 14 | censored |
| 0.05Cd, 14d | 23 | 14 | censored |
| 0.05Cd, 14d | 24 | 14 | censored |
| 0.05Cd, 14d | 25 | 14 | censored |
| 0.05Cd, 14d | 26 | 14 | censored |
| 0.05Cd, 14d | 27 | 14 | censored |
| 0.05Cd, 14d | 28 | 14 | censored |
| 0.05Cd, 14d | 29 | 14 | censored |
| 0.05Cd, 14d | 30 | 14 | censored |
| 0.05Cd, 14d | 31 | 14 | censored |
| 0.05Cd, 14d | 32 | 14 | censored |
| 0.05Cd, 14d | 33 | 14 | censored |
| 0.05Cd, 14d | 34 | 14 | censored |
| 0.05Cd, 14d | 35 | 14 | censored |
| 0.05Cd, 14d | 36 | 14 | censored |
| 0.05Cd, 14d | 37 | 14 | censored |
| 0.05Cd, 14d | 38 | 14 | censored |
| 0.05Cd, 14d | 39 | 14 | censored |
| 0.05Cd, 14d | 40 | 14 | censored |
| 0.05Cd, 14d | 41 | 14 | censored |
| 0.05Cd, 14d | 42 | 14 | censored |
| 0.05Cd, 14d | 43 | 14 | censored |
| 0.05Cd, 14d | 44 | 14 | censored |
| 0.05Cd, 14d | 45 | 14 | censored |
| 0.05Cd, 14d | 46 | 14 | censored |
| 0.05Cd, 14d | 47 | 14 | censored |
| 0.05Cd, 14d | 48 | 14 | censored |
| 0.05Cd, 14d | 49 | 14 | censored |
| 0.05Cd, 14d | 50 | 14 | censored |
| 0.05Cd, 14d | 51 | 14 | censored |
| 0.05Cd, 14d | 52 | 14 | censored |
| 0.05Cd, 14d | 53 | 14 | censored |
| 0.05Cd, 14d | 54 | 14 | censored |
| 0.05Cd, 14d | 55 | 14 | censored |
| 0.05Cd, 14d | 56 | 14 | censored |
| 0.05Cd, 14d | 57 | 14 | censored |
| 0.05Cd, 14d | 58 | 14 | censored |
| 0.05Cd, 14d | 59 | 14 | censored |
| 0.05Cd, 14d | 60 | 14 | censored |
| 0.05Cd, 14d | 61 | 14 | censored |
| 0.05Cd, 14d | 62 | 14 | censored |
| 0.05Cd, 14d | 63 | 14 | censored |
| 0.05Cd, 14d | 64 | 14 | censored |
| 0.05Cd, 14d | 65 | 14 | censored |
| 0.05Cd, 14d | 66 | 14 | censored |
| 0.05Cd, 14d | 67 | 14 | censored |
| 0.05Cd, 14d | 68 | 14 | censored |
| 0.05Cd, 14d | 69 | 14 | censored |
| 0.05Cd, 14d | 70 | 14 | censored |
| 0.05Cd, 14d | 71 | 14 | censored |
| 0.05Cd, 14d | 72 | 14 | censored |
| 0.05Cd, 14d | 73 | 14 | censored |
| 0.05Cd, 14d | 74 | 14 | censored |
| 0.05Cd, 14d | 75 | 14 | censored |
| 0.0Cd, 14d | 1 | 3 | complete |
| 0.0Cd, 14d | 2 | 9 | complete |
| 0.0Cd, 14d | 3 | 11 | complete |
| 0.0Cd, 14d | 4 | 13 | complete |
| 0.0Cd, 14d | 5 | 15 | complete |
| 0.0Cd, 14d | 6 | 14 | censored |
| 0.0Cd, 14d | 7 | 14 | censored |
| 0.0Cd, 14d | 8 | 14 | censored |
| 0.0Cd, 14d | 9 | 14 | censored |
| 0.0Cd, 14d | 10 | 14 | censored |
| 0.0Cd, 14d | 11 | 14 | censored |
| 0.0Cd, 14d | 12 | 14 | censored |
| 0.0Cd, 14d | 13 | 14 | censored |
| 0.0Cd, 14d | 14 | 14 | censored |
| 0.0Cd, 14d | 15 | 14 | censored |
| 0.0Cd, 14d | 16 | 14 | censored |
| 0.0Cd, 14d | 17 | 14 | censored |
| 0.0Cd, 14d | 18 | 14 | censored |
| 0.0Cd, 14d | 19 | 14 | censored |
| 0.0Cd, 14d | 20 | 14 | censored |
| 0.0Cd, 14d | 21 | 14 | censored |
| 0.0Cd, 14d | 22 | 14 | censored |
| 0.0Cd, 14d | 23 | 14 | censored |
| 0.0Cd, 14d | 24 | 14 | censored |
| 0.0Cd, 14d | 25 | 14 | censored |
| 0.0Cd, 14d | 26 | 14 | censored |
| 0.0Cd, 14d | 27 | 14 | censored |
| 0.0Cd, 14d | 28 | 14 | censored |
| 0.0Cd, 14d | 29 | 14 | censored |
| 0.0Cd, 14d | 30 | 14 | censored |
| 0.0Cd, 14d | 31 | 14 | censored |
| 0.0Cd, 14d | 32 | 14 | censored |
| 0.0Cd, 14d | 33 | 14 | censored |
| 0.0Cd, 14d | 34 | 14 | censored |
| 0.0Cd, 14d | 35 | 14 | censored |
| 0.0Cd, 14d | 36 | 14 | censored |
| 0.0Cd, 14d | 37 | 14 | censored |
| 0.0Cd, 14d | 38 | 14 | censored |
| 0.0Cd, 14d | 39 | 14 | censored |
| 0.0Cd, 14d | 40 | 14 | censored |
| 0.0Cd, 14d | 41 | 14 | censored |
| 0.0Cd, 14d | 42 | 14 | censored |
| 0.0Cd, 14d | 43 | 14 | censored |
| 0.0Cd, 14d | 44 | 14 | censored |
| 0.0Cd, 14d | 45 | 14 | censored |
| 0.0Cd, 14d | 46 | 14 | censored |
| 0.0Cd, 14d | 47 | 14 | censored |
| 0.0Cd, 14d | 48 | 14 | censored |
| 0.0Cd, 14d | 49 | 14 | censored |
| 0.0Cd, 14d | 50 | 14 | censored |
| 0.0Cd, 14d | 51 | 14 | censored |
| 0.0Cd, 14d | 52 | 14 | censored |
| 0.0Cd, 14d | 53 | 14 | censored |
| 0.0Cd, 14d | 54 | 14 | censored |
| 0.0Cd, 14d | 55 | 14 | censored |
| 0.0Cd, 14d | 56 | 14 | censored |
| 0.0Cd, 14d | 57 | 14 | censored |
| 0.0Cd, 14d | 58 | 14 | censored |
| 0.0Cd, 14d | 59 | 14 | censored |
| 0.0Cd, 14d | 60 | 14 | censored |
| 0.0Cd, 14d | 61 | 14 | censored |
| 0.0Cd, 14d | 62 | 14 | censored |
| 0.0Cd, 14d | 63 | 14 | censored |
| 0.0Cd, 14d | 64 | 14 | censored |
| 0.0Cd, 14d | 65 | 14 | censored |
| 0.0Cd, 14d | 66 | 14 | censored |
| 0.0Cd, 14d | 67 | 14 | censored |
| 0.0Cd, 14d | 68 | 14 | censored |
| 0.0Cd, 14d | 69 | 14 | censored |
| 0.0Cd, 14d | 70 | 14 | censored |
| 0.0Cd, 14d | 71 | 14 | censored |
| 0.0Cd, 14d | 72 | 14 | censored |
| 0.0Cd, 14d | 73 | 14 | censored |
| 0.0Cd, 14d | 74 | 14 | censored |
| 0.0Cd, 14d | 75 | 14 | censored |
| 100Cd, 28d | 1 | 19 | complete |
| 100Cd, 28d | 2 | 22 | complete |
| 100Cd, 28d | 3 | 22 | complete |
| 100Cd, 28d | 4 | 23 | complete |
| 100Cd, 28d | 5 | 25 | complete |
| 100Cd, 28d | 6 | 25 | complete |
| 100Cd, 28d | 7 | 25 | complete |
| 100Cd, 28d | 8 | 28 | censored |
| 100Cd, 28d | 9 | 28 | censored |
| 100Cd, 28d | 10 | 28 | censored |
| 100Cd, 28d | 11 | 28 | censored |
| 100Cd, 28d | 12 | 28 | censored |
| 100Cd, 28d | 13 | 28 | censored |
| 100Cd, 28d | 14 | 28 | censored |
| 100Cd, 28d | 15 | 28 | censored |
| 100Cd, 28d | 16 | 28 | censored |
| 100Cd, 28d | 17 | 28 | censored |
| 100Cd, 28d | 18 | 28 | censored |
| 100Cd, 28d | 19 | 28 | censored |
| 100Cd, 28d | 20 | 28 | censored |
| 100Cd, 28d | 21 | 28 | censored |
| 100Cd, 28d | 22 | 28 | censored |
| 100Cd, 28d | 23 | 28 | censored |
| 100Cd, 28d | 24 | 28 | censored |
| 100Cd, 28d | 25 | 28 | censored |
| 100Cd, 28d | 26 | 28 | censored |
| 100Cd, 28d | 27 | 28 | censored |
| 100Cd, 28d | 28 | 28 | censored |
| 100Cd, 28d | 29 | 28 | censored |
| 100Cd, 28d | 30 | 28 | censored |
| 100Cd, 28d | 31 | 28 | censored |
| 100Cd, 28d | 32 | 28 | censored |
| 100Cd, 28d | 33 | 28 | censored |
| 100Cd, 28d | 34 | 28 | censored |
| 100Cd, 28d | 35 | 28 | censored |
| 100Cd, 28d | 36 | 28 | censored |
| 100Cd, 28d | 37 | 28 | censored |
| 100Cd, 28d | 38 | 28 | censored |
| 100Cd, 28d | 39 | 28 | censored |
| 100Cd, 28d | 40 | 28 | censored |
| 100Cd, 28d | 41 | 28 | censored |
| 100Cd, 28d | 42 | 28 | censored |
| 100Cd, 28d | 43 | 28 | censored |
| 100Cd, 28d | 44 | 28 | censored |
| 100Cd, 28d | 45 | 28 | censored |
| 100Cd, 28d | 46 | 28 | censored |
| 100Cd, 28d | 47 | 28 | censored |
| 100Cd, 28d | 48 | 28 | censored |
| 100Cd, 28d | 49 | 28 | censored |
| 100Cd, 28d | 50 | 28 | censored |
| 100Cd, 28d | 51 | 28 | censored |
| 100Cd, 28d | 52 | 28 | censored |
| 100Cd, 28d | 53 | 28 | censored |
| 100Cd, 28d | 54 | 28 | censored |
| 100Cd, 28d | 55 | 28 | censored |
| 100Cd, 28d | 56 | 28 | censored |
| 100Cd, 28d | 57 | 28 | censored |
| 100Cd, 28d | 58 | 28 | censored |
| 100Cd, 28d | 59 | 28 | censored |
| 100Cd, 28d | 60 | 28 | censored |
| 100Cd, 28d | 61 | 28 | censored |
| 100Cd, 28d | 62 | 28 | censored |
| 10Cd, 28d | 1 | 20 | complete |
| 10Cd, 28d | 2 | 22 | complete |
| 10Cd, 28d | 3 | 23 | complete |
| 10Cd, 28d | 4 | 24 | complete |
| 10Cd, 28d | 5 | 25 | complete |
| 10Cd, 28d | 6 | 28 | censored |
| 10Cd, 28d | 7 | 28 | censored |
| 10Cd, 28d | 8 | 28 | censored |
| 10Cd, 28d | 9 | 28 | censored |
| 10Cd, 28d | 10 | 28 | censored |
| 10Cd, 28d | 11 | 28 | censored |
| 10Cd, 28d | 12 | 28 | censored |
| 10Cd, 28d | 13 | 28 | censored |
| 10Cd, 28d | 14 | 28 | censored |
| 10Cd, 28d | 15 | 28 | censored |
| 10Cd, 28d | 16 | 28 | censored |
| 10Cd, 28d | 17 | 28 | censored |
| 10Cd, 28d | 18 | 28 | censored |
| 10Cd, 28d | 19 | 28 | censored |
| 10Cd, 28d | 20 | 28 | censored |
| 10Cd, 28d | 21 | 28 | censored |
| 10Cd, 28d | 22 | 28 | censored |
| 10Cd, 28d | 23 | 28 | censored |
| 10Cd, 28d | 24 | 28 | censored |
| 10Cd, 28d | 25 | 28 | censored |
| 10Cd, 28d | 26 | 28 | censored |
| 10Cd, 28d | 27 | 28 | censored |
| 10Cd, 28d | 28 | 28 | censored |
| 10Cd, 28d | 29 | 28 | censored |
| 10Cd, 28d | 30 | 28 | censored |
| 10Cd, 28d | 31 | 28 | censored |
| 10Cd, 28d | 32 | 28 | censored |
| 10Cd, 28d | 33 | 28 | censored |
| 10Cd, 28d | 34 | 28 | censored |
| 10Cd, 28d | 35 | 28 | censored |
| 10Cd, 28d | 36 | 28 | censored |
| 10Cd, 28d | 37 | 28 | censored |
| 10Cd, 28d | 38 | 28 | censored |
| 10Cd, 28d | 39 | 28 | censored |
| 10Cd, 28d | 40 | 28 | censored |
| 10Cd, 28d | 41 | 28 | censored |
| 10Cd, 28d | 42 | 28 | censored |
| 10Cd, 28d | 43 | 28 | censored |
| 10Cd, 28d | 44 | 28 | censored |
| 10Cd, 28d | 45 | 28 | censored |
| 10Cd, 28d | 46 | 28 | censored |
| 10Cd, 28d | 47 | 28 | censored |
| 10Cd, 28d | 48 | 28 | censored |
| 10Cd, 28d | 49 | 28 | censored |
| 10Cd, 28d | 50 | 28 | censored |
| 10Cd, 28d | 51 | 28 | censored |
| 10Cd, 28d | 52 | 28 | censored |
| 10Cd, 28d | 53 | 28 | censored |
| 10Cd, 28d | 54 | 28 | censored |
| 10Cd, 28d | 55 | 28 | censored |
| 10Cd, 28d | 56 | 28 | censored |
| 10Cd, 28d | 57 | 28 | censored |
| 10Cd, 28d | 58 | 28 | censored |
| 10Cd, 28d | 59 | 28 | censored |
| 10Cd, 28d | 60 | 28 | censored |
| 10Cd, 28d | 61 | 28 | censored |
| 10Cd, 28d | 62 | 28 | censored |
| 10Cd, 28d | 63 | 28 | censored |
| 1Cd, 28d | 1 | 16 | complete |
| 1Cd, 28d | 2 | 16 | complete |
| 1Cd, 28d | 3 | 20 | complete |
| 1Cd, 28d | 4 | 22 | complete |
| 1Cd, 28d | 5 | 22 | complete |
| 1Cd, 28d | 6 | 28 | censored |
| 1Cd, 28d | 7 | 28 | censored |
| 1Cd, 28d | 8 | 28 | censored |
| 1Cd, 28d | 9 | 28 | censored |
| 1Cd, 28d | 10 | 28 | censored |
| 1Cd, 28d | 11 | 28 | censored |
| 1Cd, 28d | 12 | 28 | censored |
| 1Cd, 28d | 13 | 28 | censored |
| 1Cd, 28d | 14 | 28 | censored |
| 1Cd, 28d | 15 | 28 | censored |
| 1Cd, 28d | 16 | 28 | censored |
| 1Cd, 28d | 17 | 28 | censored |
| 1Cd, 28d | 18 | 28 | censored |
| 1Cd, 28d | 19 | 28 | censored |
| 1Cd, 28d | 20 | 28 | censored |
| 1Cd, 28d | 21 | 28 | censored |
| 1Cd, 28d | 22 | 28 | censored |
| 1Cd, 28d | 23 | 28 | censored |
| 1Cd, 28d | 24 | 28 | censored |
| 1Cd, 28d | 25 | 28 | censored |
| 1Cd, 28d | 26 | 28 | censored |
| 1Cd, 28d | 27 | 28 | censored |
| 1Cd, 28d | 28 | 28 | censored |
| 1Cd, 28d | 29 | 28 | censored |
| 1Cd, 28d | 30 | 28 | censored |
| 1Cd, 28d | 31 | 28 | censored |
| 1Cd, 28d | 32 | 28 | censored |
| 1Cd, 28d | 33 | 28 | censored |
| 1Cd, 28d | 34 | 28 | censored |
| 1Cd, 28d | 35 | 28 | censored |
| 1Cd, 28d | 36 | 28 | censored |
| 1Cd, 28d | 37 | 28 | censored |
| 1Cd, 28d | 38 | 28 | censored |
| 1Cd, 28d | 39 | 28 | censored |
| 1Cd, 28d | 40 | 28 | censored |
| 1Cd, 28d | 41 | 28 | censored |
| 1Cd, 28d | 42 | 28 | censored |
| 1Cd, 28d | 43 | 28 | censored |
| 1Cd, 28d | 44 | 28 | censored |
| 1Cd, 28d | 45 | 28 | censored |
| 1Cd, 28d | 46 | 28 | censored |
| 1Cd, 28d | 47 | 28 | censored |
| 1Cd, 28d | 48 | 28 | censored |
| 1Cd, 28d | 49 | 28 | censored |
| 1Cd, 28d | 50 | 28 | censored |
| 1Cd, 28d | 51 | 28 | censored |
| 1Cd, 28d | 52 | 28 | censored |
| 1Cd, 28d | 53 | 28 | censored |
| 1Cd, 28d | 54 | 28 | censored |
| 1Cd, 28d | 55 | 28 | censored |
| 1Cd, 28d | 56 | 28 | censored |
| 1Cd, 28d | 57 | 28 | censored |
| 1Cd, 28d | 58 | 28 | censored |
| 1Cd, 28d | 59 | 28 | censored |
| 1Cd, 28d | 60 | 28 | censored |
| 1Cd, 28d | 61 | 28 | censored |
| 1Cd, 28d | 62 | 28 | censored |
| 1Cd, 28d | 63 | 28 | censored |
| 0.2Cd, 28d | 1 | 16 | complete |
| 0.2Cd, 28d | 2 | 20 | complete |
| 0.2Cd, 28d | 3 | 24 | complete |
| 0.2Cd, 28d | 4 | 28 | complete |
| 0.2Cd, 28d | 5 | 28 | censored |
| 0.2Cd, 28d | 6 | 28 | censored |
| 0.2Cd, 28d | 7 | 28 | censored |
| 0.2Cd, 28d | 8 | 28 | censored |
| 0.2Cd, 28d | 9 | 28 | censored |
| 0.2Cd, 28d | 10 | 28 | censored |
| 0.2Cd, 28d | 11 | 28 | censored |
| 0.2Cd, 28d | 12 | 28 | censored |
| 0.2Cd, 28d | 13 | 28 | censored |
| 0.2Cd, 28d | 14 | 28 | censored |
| 0.2Cd, 28d | 15 | 28 | censored |
| 0.2Cd, 28d | 16 | 28 | censored |
| 0.2Cd, 28d | 17 | 28 | censored |
| 0.2Cd, 28d | 18 | 28 | censored |
| 0.2Cd, 28d | 19 | 28 | censored |
| 0.2Cd, 28d | 20 | 28 | censored |
| 0.2Cd, 28d | 21 | 28 | censored |
| 0.2Cd, 28d | 22 | 28 | censored |
| 0.2Cd, 28d | 23 | 28 | censored |
| 0.2Cd, 28d | 24 | 28 | censored |
| 0.2Cd, 28d | 25 | 28 | censored |
| 0.2Cd, 28d | 26 | 28 | censored |
| 0.2Cd, 28d | 27 | 28 | censored |
| 0.2Cd, 28d | 28 | 28 | censored |
| 0.2Cd, 28d | 29 | 28 | censored |
| 0.2Cd, 28d | 30 | 28 | censored |
| 0.2Cd, 28d | 31 | 28 | censored |
| 0.2Cd, 28d | 32 | 28 | censored |
| 0.2Cd, 28d | 33 | 28 | censored |
| 0.2Cd, 28d | 34 | 28 | censored |
| 0.2Cd, 28d | 35 | 28 | censored |
| 0.2Cd, 28d | 36 | 28 | censored |
| 0.2Cd, 28d | 37 | 28 | censored |
| 0.2Cd, 28d | 38 | 28 | censored |
| 0.2Cd, 28d | 39 | 28 | censored |
| 0.2Cd, 28d | 40 | 28 | censored |
| 0.2Cd, 28d | 41 | 28 | censored |
| 0.2Cd, 28d | 42 | 28 | censored |
| 0.2Cd, 28d | 43 | 28 | censored |
| 0.2Cd, 28d | 44 | 28 | censored |
| 0.2Cd, 28d | 45 | 28 | censored |
| 0.2Cd, 28d | 46 | 28 | censored |
| 0.2Cd, 28d | 47 | 28 | censored |
| 0.2Cd, 28d | 48 | 28 | censored |
| 0.2Cd, 28d | 49 | 28 | censored |
| 0.2Cd, 28d | 50 | 28 | censored |
| 0.2Cd, 28d | 51 | 28 | censored |
| 0.2Cd, 28d | 52 | 28 | censored |
| 0.2Cd, 28d | 53 | 28 | censored |
| 0.2Cd, 28d | 54 | 28 | censored |
| 0.2Cd, 28d | 55 | 28 | censored |
| 0.2Cd, 28d | 56 | 28 | censored |
| 0.2Cd, 28d | 57 | 28 | censored |
| 0.2Cd, 28d | 58 | 28 | censored |
| 0.2Cd, 28d | 59 | 28 | censored |
| 0.2Cd, 28d | 60 | 28 | censored |
| 0.2Cd, 28d | 61 | 28 | censored |
| 0.2Cd, 28d | 62 | 28 | censored |
| 0.2Cd, 28d | 63 | 28 | censored |
| 0.2Cd, 28d | 64 | 28 | censored |
| 0.05Cd, 28d | 1 | 17 | complete |
| 0.05Cd, 28d | 2 | 20 | complete |
| 0.05Cd, 28d | 3 | 25 | complete |
| 0.05Cd, 28d | 4 | 28 | censored |
| 0.05Cd, 28d | 5 | 28 | censored |
| 0.05Cd, 28d | 6 | 28 | censored |
| 0.05Cd, 28d | 7 | 28 | censored |
| 0.05Cd, 28d | 8 | 28 | censored |
| 0.05Cd, 28d | 9 | 28 | censored |
| 0.05Cd, 28d | 10 | 28 | censored |
| 0.05Cd, 28d | 11 | 28 | censored |
| 0.05Cd, 28d | 12 | 28 | censored |
| 0.05Cd, 28d | 13 | 28 | censored |
| 0.05Cd, 28d | 14 | 28 | censored |
| 0.05Cd, 28d | 15 | 28 | censored |
| 0.05Cd, 28d | 16 | 28 | censored |
| 0.05Cd, 28d | 17 | 28 | censored |
| 0.05Cd, 28d | 18 | 28 | censored |
| 0.05Cd, 28d | 19 | 28 | censored |
| 0.05Cd, 28d | 20 | 28 | censored |
| 0.05Cd, 28d | 21 | 28 | censored |
| 0.05Cd, 28d | 22 | 28 | censored |
| 0.05Cd, 28d | 23 | 28 | censored |
| 0.05Cd, 28d | 24 | 28 | censored |
| 0.05Cd, 28d | 25 | 28 | censored |
| 0.05Cd, 28d | 26 | 28 | censored |
| 0.05Cd, 28d | 27 | 28 | censored |
| 0.05Cd, 28d | 28 | 28 | censored |
| 0.05Cd, 28d | 29 | 28 | censored |
| 0.05Cd, 28d | 30 | 28 | censored |
| 0.05Cd, 28d | 31 | 28 | censored |
| 0.05Cd, 28d | 32 | 28 | censored |
| 0.05Cd, 28d | 33 | 28 | censored |
| 0.05Cd, 28d | 34 | 28 | censored |
| 0.05Cd, 28d | 35 | 28 | censored |
| 0.05Cd, 28d | 36 | 28 | censored |
| 0.05Cd, 28d | 37 | 28 | censored |
| 0.05Cd, 28d | 38 | 28 | censored |
| 0.05Cd, 28d | 39 | 28 | censored |
| 0.05Cd, 28d | 40 | 28 | censored |
| 0.05Cd, 28d | 41 | 28 | censored |
| 0.05Cd, 28d | 42 | 28 | censored |
| 0.05Cd, 28d | 43 | 28 | censored |
| 0.05Cd, 28d | 44 | 28 | censored |
| 0.05Cd, 28d | 45 | 28 | censored |
| 0.05Cd, 28d | 46 | 28 | censored |
| 0.05Cd, 28d | 47 | 28 | censored |
| 0.05Cd, 28d | 48 | 28 | censored |
| 0.05Cd, 28d | 49 | 28 | censored |
| 0.05Cd, 28d | 50 | 28 | censored |
| 0.05Cd, 28d | 51 | 28 | censored |
| 0.05Cd, 28d | 52 | 28 | censored |
| 0.05Cd, 28d | 53 | 28 | censored |
| 0.05Cd, 28d | 54 | 28 | censored |
| 0.05Cd, 28d | 55 | 28 | censored |
| 0.05Cd, 28d | 56 | 28 | censored |
| 0.05Cd, 28d | 57 | 28 | censored |
| 0.05Cd, 28d | 58 | 28 | censored |
| 0.05Cd, 28d | 59 | 28 | censored |
| 0.05Cd, 28d | 60 | 28 | censored |
| 0.05Cd, 28d | 61 | 28 | censored |
| 0.05Cd, 28d | 62 | 28 | censored |
| 0.05Cd, 28d | 63 | 28 | censored |
| 0.05Cd, 28d | 64 | 28 | censored |
| 0.05Cd, 28d | 65 | 28 | censored |
| 0.0Cd, 28d | 1 | 20 | complete |
| 0.0Cd, 28d | 2 | 25 | complete |
| 0.0Cd, 28d | 3 | 26 | complete |
| 0.0Cd, 28d | 4 | 27 | complete |
| 0.0Cd, 28d | 5 | 28 | censored |
| 0.0Cd, 28d | 6 | 28 | censored |
| 0.0Cd, 28d | 7 | 28 | censored |
| 0.0Cd, 28d | 8 | 28 | censored |
| 0.0Cd, 28d | 9 | 28 | censored |
| 0.0Cd, 28d | 10 | 28 | censored |
| 0.0Cd, 28d | 11 | 28 | censored |
| 0.0Cd, 28d | 12 | 28 | censored |
| 0.0Cd, 28d | 13 | 28 | censored |
| 0.0Cd, 28d | 14 | 28 | censored |
| 0.0Cd, 28d | 15 | 28 | censored |
| 0.0Cd, 28d | 16 | 28 | censored |
| 0.0Cd, 28d | 17 | 28 | censored |
| 0.0Cd, 28d | 18 | 28 | censored |
| 0.0Cd, 28d | 19 | 28 | censored |
| 0.0Cd, 28d | 20 | 28 | censored |
| 0.0Cd, 28d | 21 | 28 | censored |
| 0.0Cd, 28d | 22 | 28 | censored |
| 0.0Cd, 28d | 23 | 28 | censored |
| 0.0Cd, 28d | 24 | 28 | censored |
| 0.0Cd, 28d | 25 | 28 | censored |
| 0.0Cd, 28d | 26 | 28 | censored |
| 0.0Cd, 28d | 27 | 28 | censored |
| 0.0Cd, 28d | 28 | 28 | censored |
| 0.0Cd, 28d | 29 | 28 | censored |
| 0.0Cd, 28d | 30 | 28 | censored |
| 0.0Cd, 28d | 31 | 28 | censored |
| 0.0Cd, 28d | 32 | 28 | censored |
| 0.0Cd, 28d | 33 | 28 | censored |
| 0.0Cd, 28d | 34 | 28 | censored |
| 0.0Cd, 28d | 35 | 28 | censored |
| 0.0Cd, 28d | 36 | 28 | censored |
| 0.0Cd, 28d | 37 | 28 | censored |
| 0.0Cd, 28d | 38 | 28 | censored |
| 0.0Cd, 28d | 39 | 28 | censored |
| 0.0Cd, 28d | 40 | 28 | censored |
| 0.0Cd, 28d | 41 | 28 | censored |
| 0.0Cd, 28d | 42 | 28 | censored |
| 0.0Cd, 28d | 43 | 28 | censored |
| 0.0Cd, 28d | 44 | 28 | censored |
| 0.0Cd, 28d | 45 | 28 | censored |
| 0.0Cd, 28d | 46 | 28 | censored |
| 0.0Cd, 28d | 47 | 28 | censored |
| 0.0Cd, 28d | 48 | 28 | censored |
| 0.0Cd, 28d | 49 | 28 | censored |
| 0.0Cd, 28d | 50 | 28 | censored |
| 0.0Cd, 28d | 51 | 28 | censored |
| 0.0Cd, 28d | 52 | 28 | censored |
| 0.0Cd, 28d | 53 | 28 | censored |
| 0.0Cd, 28d | 54 | 28 | censored |
| 0.0Cd, 28d | 55 | 28 | censored |
| 0.0Cd, 28d | 56 | 28 | censored |
| 0.0Cd, 28d | 57 | 28 | censored |
| 0.0Cd, 28d | 58 | 28 | censored |
| 0.0Cd, 28d | 59 | 28 | censored |
| 0.0Cd, 28d | 60 | 28 | censored |
| 0.0Cd, 28d | 61 | 28 | censored |
| 0.0Cd, 28d | 62 | 28 | censored |
| 0.0Cd, 28d | 63 | 28 | censored |
| 0.0Cd, 28d | 64 | 28 | censored |
| 100Cd, 56d | 1 | 29 | complete |
| 100Cd, 56d | 2 | 34 | complete |
| 100Cd, 56d | 3 | 35 | complete |
| 100Cd, 56d | 4 | 45 | complete |
| 100Cd, 56d | 5 | 51 | complete |
| 100Cd, 56d | 6 | 53 | complete |
| 100Cd, 56d | 7 | 56 | censored |
| 100Cd, 56d | 8 | 56 | censored |
| 100Cd, 56d | 9 | 56 | censored |
| 100Cd, 56d | 10 | 56 | censored |
| 100Cd, 56d | 11 | 56 | censored |
| 100Cd, 56d | 12 | 56 | censored |
| 100Cd, 56d | 13 | 56 | censored |
| 100Cd, 56d | 14 | 56 | censored |
| 100Cd, 56d | 15 | 56 | censored |
| 100Cd, 56d | 16 | 56 | censored |
| 100Cd, 56d | 17 | 56 | censored |
| 100Cd, 56d | 18 | 56 | censored |
| 100Cd, 56d | 19 | 56 | censored |
| 100Cd, 56d | 20 | 56 | censored |
| 100Cd, 56d | 21 | 56 | censored |
| 100Cd, 56d | 22 | 56 | censored |
| 100Cd, 56d | 23 | 56 | censored |
| 100Cd, 56d | 24 | 56 | censored |
| 100Cd, 56d | 25 | 56 | censored |
| 100Cd, 56d | 26 | 56 | censored |
| 100Cd, 56d | 27 | 56 | censored |
| 100Cd, 56d | 28 | 56 | censored |
| 100Cd, 56d | 29 | 56 | censored |
| 100Cd, 56d | 30 | 56 | censored |
| 100Cd, 56d | 31 | 56 | censored |
| 100Cd, 56d | 32 | 56 | censored |
| 100Cd, 56d | 33 | 56 | censored |
| 100Cd, 56d | 34 | 56 | censored |
| 100Cd, 56d | 35 | 56 | censored |
| 100Cd, 56d | 36 | 56 | censored |
| 100Cd, 56d | 37 | 56 | censored |
| 100Cd, 56d | 38 | 56 | censored |
| 100Cd, 56d | 39 | 56 | censored |
| 100Cd, 56d | 40 | 56 | censored |
| 100Cd, 56d | 41 | 56 | censored |
| 100Cd, 56d | 42 | 56 | censored |
| 100Cd, 56d | 43 | 56 | censored |
| 100Cd, 56d | 44 | 56 | censored |
| 100Cd, 56d | 45 | 56 | censored |
| 100Cd, 56d | 46 | 56 | censored |
| 100Cd, 56d | 47 | 56 | censored |
| 100Cd, 56d | 48 | 56 | censored |
| 100Cd, 56d | 49 | 56 | censored |
| 10Cd, 56d | 1 | 32 | complete |
| 10Cd, 56d | 2 | 40 | complete |
| 10Cd, 56d | 3 | 51 | complete |
| 10Cd, 56d | 4 | 52 | complete |
| 10Cd, 56d | 5 | 56 | censored |
| 10Cd, 56d | 6 | 56 | censored |
| 10Cd, 56d | 7 | 56 | censored |
| 10Cd, 56d | 8 | 56 | censored |
| 10Cd, 56d | 9 | 56 | censored |
| 10Cd, 56d | 10 | 56 | censored |
| 10Cd, 56d | 11 | 56 | censored |
| 10Cd, 56d | 12 | 56 | censored |
| 10Cd, 56d | 13 | 56 | censored |
| 10Cd, 56d | 14 | 56 | censored |
| 10Cd, 56d | 15 | 56 | censored |
| 10Cd, 56d | 16 | 56 | censored |
| 10Cd, 56d | 17 | 56 | censored |
| 10Cd, 56d | 18 | 56 | censored |
| 10Cd, 56d | 19 | 56 | censored |
| 10Cd, 56d | 20 | 56 | censored |
| 10Cd, 56d | 21 | 56 | censored |
| 10Cd, 56d | 22 | 56 | censored |
| 10Cd, 56d | 23 | 56 | censored |
| 10Cd, 56d | 24 | 56 | censored |
| 10Cd, 56d | 25 | 56 | censored |
| 10Cd, 56d | 26 | 56 | censored |
| 10Cd, 56d | 27 | 56 | censored |
| 10Cd, 56d | 28 | 56 | censored |
| 10Cd, 56d | 29 | 56 | censored |
| 10Cd, 56d | 30 | 56 | censored |
| 10Cd, 56d | 31 | 56 | censored |
| 10Cd, 56d | 32 | 56 | censored |
| 10Cd, 56d | 33 | 56 | censored |
| 10Cd, 56d | 34 | 56 | censored |
| 10Cd, 56d | 35 | 56 | censored |
| 10Cd, 56d | 36 | 56 | censored |
| 10Cd, 56d | 37 | 56 | censored |
| 10Cd, 56d | 38 | 56 | censored |
| 10Cd, 56d | 39 | 56 | censored |
| 10Cd, 56d | 40 | 56 | censored |
| 10Cd, 56d | 41 | 56 | censored |
| 10Cd, 56d | 42 | 56 | censored |
| 10Cd, 56d | 43 | 56 | censored |
| 10Cd, 56d | 44 | 56 | censored |
| 10Cd, 56d | 45 | 56 | censored |
| 10Cd, 56d | 46 | 56 | censored |
| 10Cd, 56d | 47 | 56 | censored |
| 10Cd, 56d | 48 | 56 | censored |
| 10Cd, 56d | 49 | 56 | censored |
| 10Cd, 56d | 50 | 56 | censored |
| 10Cd, 56d | 51 | 56 | censored |
| 10Cd, 56d | 52 | 56 | censored |
| 1Cd, 56d | 1 | 32 | complete |
| 1Cd, 56d | 2 | 34 | complete |
| 1Cd, 56d | 3 | 40 | complete |
| 1Cd, 56d | 4 | 42 | complete |
| 1Cd, 56d | 5 | 46 | complete |
| 1Cd, 56d | 6 | 49 | complete |
| 1Cd, 56d | 7 | 53 | complete |
| 1Cd, 56d | 8 | 56 | censored |
| 1Cd, 56d | 9 | 56 | censored |
| 1Cd, 56d | 10 | 56 | censored |
| 1Cd, 56d | 11 | 56 | censored |
| 1Cd, 56d | 12 | 56 | censored |
| 1Cd, 56d | 13 | 56 | censored |
| 1Cd, 56d | 14 | 56 | censored |
| 1Cd, 56d | 15 | 56 | censored |
| 1Cd, 56d | 16 | 56 | censored |
| 1Cd, 56d | 17 | 56 | censored |
| 1Cd, 56d | 18 | 56 | censored |
| 1Cd, 56d | 19 | 56 | censored |
| 1Cd, 56d | 20 | 56 | censored |
| 1Cd, 56d | 21 | 56 | censored |
| 1Cd, 56d | 22 | 56 | censored |
| 1Cd, 56d | 23 | 56 | censored |
| 1Cd, 56d | 24 | 56 | censored |
| 1Cd, 56d | 25 | 56 | censored |
| 1Cd, 56d | 26 | 56 | censored |
| 1Cd, 56d | 27 | 56 | censored |
| 1Cd, 56d | 28 | 56 | censored |
| 1Cd, 56d | 29 | 56 | censored |
| 1Cd, 56d | 30 | 56 | censored |
| 1Cd, 56d | 31 | 56 | censored |
| 1Cd, 56d | 32 | 56 | censored |
| 1Cd, 56d | 33 | 56 | censored |
| 1Cd, 56d | 34 | 56 | censored |
| 1Cd, 56d | 35 | 56 | censored |
| 1Cd, 56d | 36 | 56 | censored |
| 1Cd, 56d | 37 | 56 | censored |
| 1Cd, 56d | 38 | 56 | censored |
| 1Cd, 56d | 39 | 56 | censored |
| 1Cd, 56d | 40 | 56 | censored |
| 1Cd, 56d | 41 | 56 | censored |
| 1Cd, 56d | 42 | 56 | censored |
| 1Cd, 56d | 43 | 56 | censored |
| 1Cd, 56d | 44 | 56 | censored |
| 1Cd, 56d | 45 | 56 | censored |
| 1Cd, 56d | 46 | 56 | censored |
| 1Cd, 56d | 47 | 56 | censored |
| 1Cd, 56d | 48 | 56 | censored |
| 1Cd, 56d | 49 | 56 | censored |
| 1Cd, 56d | 50 | 56 | censored |
| 1Cd, 56d | 51 | 56 | censored |
| 1Cd, 56d | 52 | 56 | censored |
| 0.2Cd, 56d | 1 | 32 | complete |
| 0.2Cd, 56d | 2 | 34 | complete |
| 0.2Cd, 56d | 3 | 40 | complete |
| 0.2Cd, 56d | 4 | 42 | complete |
| 0.2Cd, 56d | 5 | 46 | complete |
| 0.2Cd, 56d | 6 | 49 | complete |
| 0.2Cd, 56d | 7 | 53 | complete |
| 0.2Cd, 56d | 8 | 52 | complete |
| 0.2Cd, 56d | 9 | 53 | complete |
| 0.2Cd, 56d | 10 | 56 | censored |
| 0.2Cd, 56d | 11 | 56 | censored |
| 0.2Cd, 56d | 12 | 56 | censored |
| 0.2Cd, 56d | 13 | 56 | censored |
| 0.2Cd, 56d | 14 | 56 | censored |
| 0.2Cd, 56d | 15 | 56 | censored |
| 0.2Cd, 56d | 16 | 56 | censored |
| 0.2Cd, 56d | 17 | 56 | censored |
| 0.2Cd, 56d | 18 | 56 | censored |
| 0.2Cd, 56d | 19 | 56 | censored |
| 0.2Cd, 56d | 20 | 56 | censored |
| 0.2Cd, 56d | 21 | 56 | censored |
| 0.2Cd, 56d | 22 | 56 | censored |
| 0.2Cd, 56d | 23 | 56 | censored |
| 0.2Cd, 56d | 24 | 56 | censored |
| 0.2Cd, 56d | 25 | 56 | censored |
| 0.2Cd, 56d | 26 | 56 | censored |
| 0.2Cd, 56d | 27 | 56 | censored |
| 0.2Cd, 56d | 28 | 56 | censored |
| 0.2Cd, 56d | 29 | 56 | censored |
| 0.2Cd, 56d | 30 | 56 | censored |
| 0.2Cd, 56d | 31 | 56 | censored |
| 0.2Cd, 56d | 32 | 56 | censored |
| 0.2Cd, 56d | 33 | 56 | censored |
| 0.2Cd, 56d | 34 | 56 | censored |
| 0.2Cd, 56d | 35 | 56 | censored |
| 0.2Cd, 56d | 36 | 56 | censored |
| 0.2Cd, 56d | 37 | 56 | censored |
| 0.2Cd, 56d | 38 | 56 | censored |
| 0.2Cd, 56d | 39 | 56 | censored |
| 0.2Cd, 56d | 40 | 56 | censored |
| 0.2Cd, 56d | 41 | 56 | censored |
| 0.2Cd, 56d | 42 | 56 | censored |
| 0.2Cd, 56d | 43 | 56 | censored |
| 0.2Cd, 56d | 44 | 56 | censored |
| 0.2Cd, 56d | 45 | 56 | censored |
| 0.2Cd, 56d | 46 | 56 | censored |
| 0.2Cd, 56d | 47 | 56 | censored |
| 0.2Cd, 56d | 48 | 56 | censored |
| 0.2Cd, 56d | 49 | 56 | censored |
| 0.2Cd, 56d | 50 | 56 | censored |
| 0.2Cd, 56d | 51 | 56 | censored |
| 0.2Cd, 56d | 52 | 56 | censored |
| 0.2Cd, 56d | 53 | 56 | censored |
| 0.2Cd, 56d | 54 | 56 | censored |
| 0.05Cd, 56d | 1 | 30 | complete |
| 0.05Cd, 56d | 2 | 35 | complete |
| 0.05Cd, 56d | 3 | 49 | complete |
| 0.05Cd, 56d | 4 | 53 | complete |
| 0.05Cd, 56d | 5 | 55 | complete |
| 0.05Cd, 56d | 6 | 55 | complete |
| 0.05Cd, 56d | 7 | 56 | censored |
| 0.05Cd, 56d | 8 | 56 | censored |
| 0.05Cd, 56d | 9 | 56 | censored |
| 0.05Cd, 56d | 10 | 56 | censored |
| 0.05Cd, 56d | 11 | 56 | censored |
| 0.05Cd, 56d | 12 | 56 | censored |
| 0.05Cd, 56d | 13 | 56 | censored |
| 0.05Cd, 56d | 14 | 56 | censored |
| 0.05Cd, 56d | 15 | 56 | censored |
| 0.05Cd, 56d | 16 | 56 | censored |
| 0.05Cd, 56d | 17 | 56 | censored |
| 0.05Cd, 56d | 18 | 56 | censored |
| 0.05Cd, 56d | 19 | 56 | censored |
| 0.05Cd, 56d | 20 | 56 | censored |
| 0.05Cd, 56d | 21 | 56 | censored |
| 0.05Cd, 56d | 22 | 56 | censored |
| 0.05Cd, 56d | 23 | 56 | censored |
| 0.05Cd, 56d | 24 | 56 | censored |
| 0.05Cd, 56d | 25 | 56 | censored |
| 0.05Cd, 56d | 26 | 56 | censored |
| 0.05Cd, 56d | 27 | 56 | censored |
| 0.05Cd, 56d | 28 | 56 | censored |
| 0.05Cd, 56d | 29 | 56 | censored |
| 0.05Cd, 56d | 30 | 56 | censored |
| 0.05Cd, 56d | 31 | 56 | censored |
| 0.05Cd, 56d | 32 | 56 | censored |
| 0.05Cd, 56d | 33 | 56 | censored |
| 0.05Cd, 56d | 34 | 56 | censored |
| 0.05Cd, 56d | 35 | 56 | censored |
| 0.05Cd, 56d | 36 | 56 | censored |
| 0.05Cd, 56d | 37 | 56 | censored |
| 0.05Cd, 56d | 38 | 56 | censored |
| 0.05Cd, 56d | 39 | 56 | censored |
| 0.05Cd, 56d | 40 | 56 | censored |
| 0.05Cd, 56d | 41 | 56 | censored |
| 0.05Cd, 56d | 42 | 56 | censored |
| 0.05Cd, 56d | 43 | 56 | censored |
| 0.05Cd, 56d | 44 | 56 | censored |
| 0.05Cd, 56d | 45 | 56 | censored |
| 0.05Cd, 56d | 46 | 56 | censored |
| 0.05Cd, 56d | 47 | 56 | censored |
| 0.05Cd, 56d | 48 | 56 | censored |
| 0.05Cd, 56d | 49 | 56 | censored |
| 0.05Cd, 56d | 50 | 56 | censored |
| 0.05Cd, 56d | 51 | 56 | censored |
| 0.05Cd, 56d | 52 | 56 | censored |
| 0.05Cd, 56d | 53 | 56 | censored |
| 0.05Cd, 56d | 54 | 56 | censored |
| 0.05Cd, 56d | 55 | 56 | censored |
| 0.05Cd, 56d | 56 | 56 | censored |
| 0.0Cd, 56d | 1 | 33 | complete |
| 0.0Cd, 56d | 2 | 38 | complete |
| 0.0Cd, 56d | 3 | 43 | complete |
| 0.0Cd, 56d | 4 | 47 | complete |
| 0.0Cd, 56d | 5 | 51 | complete |
| 0.0Cd, 56d | 6 | 56 | censored |
| 0.0Cd, 56d | 7 | 56 | censored |
| 0.0Cd, 56d | 8 | 56 | censored |
| 0.0Cd, 56d | 9 | 56 | censored |
| 0.0Cd, 56d | 10 | 56 | censored |
| 0.0Cd, 56d | 11 | 56 | censored |
| 0.0Cd, 56d | 12 | 56 | censored |
| 0.0Cd, 56d | 13 | 56 | censored |
| 0.0Cd, 56d | 14 | 56 | censored |
| 0.0Cd, 56d | 15 | 56 | censored |
| 0.0Cd, 56d | 16 | 56 | censored |
| 0.0Cd, 56d | 17 | 56 | censored |
| 0.0Cd, 56d | 18 | 56 | censored |
| 0.0Cd, 56d | 19 | 56 | censored |
| 0.0Cd, 56d | 20 | 56 | censored |
| 0.0Cd, 56d | 21 | 56 | censored |
| 0.0Cd, 56d | 22 | 56 | censored |
| 0.0Cd, 56d | 23 | 56 | censored |
| 0.0Cd, 56d | 24 | 56 | censored |
| 0.0Cd, 56d | 25 | 56 | censored |
| 0.0Cd, 56d | 26 | 56 | censored |
| 0.0Cd, 56d | 27 | 56 | censored |
| 0.0Cd, 56d | 28 | 56 | censored |
| 0.0Cd, 56d | 29 | 56 | censored |
| 0.0Cd, 56d | 30 | 56 | censored |
| 0.0Cd, 56d | 31 | 56 | censored |
| 0.0Cd, 56d | 32 | 56 | censored |
| 0.0Cd, 56d | 33 | 56 | censored |
| 0.0Cd, 56d | 34 | 56 | censored |
| 0.0Cd, 56d | 35 | 56 | censored |
| 0.0Cd, 56d | 36 | 56 | censored |
| 0.0Cd, 56d | 37 | 56 | censored |
| 0.0Cd, 56d | 38 | 56 | censored |
| 0.0Cd, 56d | 39 | 56 | censored |
| 0.0Cd, 56d | 40 | 56 | censored |
| 0.0Cd, 56d | 41 | 56 | censored |
| 0.0Cd, 56d | 42 | 56 | censored |
| 0.0Cd, 56d | 43 | 56 | censored |
| 0.0Cd, 56d | 44 | 56 | censored |
| 0.0Cd, 56d | 45 | 56 | censored |
| 0.0Cd, 56d | 46 | 56 | censored |
| 0.0Cd, 56d | 47 | 56 | censored |
| 0.0Cd, 56d | 48 | 56 | censored |
| 0.0Cd, 56d | 49 | 56 | censored |
| 0.0Cd, 56d | 50 | 56 | censored |
| 0.0Cd, 56d | 51 | 56 | censored |
| 0.0Cd, 56d | 52 | 56 | censored |
| 0.0Cd, 56d | 53 | 56 | censored |
| 0.0Cd, 56d | 54 | 56 | censored |
